# Tenuivirus utilizes its glycoprotein as a helper component to overcome insect midgut barriers for its circulative and propagative transmission

**DOI:** 10.1371/journal.ppat.1007655

**Published:** 2019-03-28

**Authors:** Gang Lu, Shuo Li, Changwei Zhou, Xin Qian, Qing Xiang, Tongqing Yang, Jianxiang Wu, Xueping Zhou, Yijun Zhou, Xin S. Ding, Xiaorong Tao

**Affiliations:** 1 Department of Plant Pathology, Nanjing Agricultural University, Nanjing, P.R. China; 2 Institute of Plant Protection, Jiangsu Academy of Agricultural Sciences, Nanjing, P.R. China; 3 State Key Laboratory of Rice Biology, Institute of Biotechnology, Zhejiang University, Hangzhou, P.R. China; 4 State Key Laboratory for Biology of Plant Diseases and Insect Pests, Institute of Plant Protection, Chinese Academy of Agricultural Sciences, Beijing, P.R. China; University of California Davis, UNITED STATES

## Abstract

Many persistent transmitted plant viruses, including rice stripe virus (RSV), cause serious damage to crop production worldwide. Although many reports have indicated that a successful insect-mediated virus transmission depends on a proper interaction between the virus and its insect vector, the mechanism(s) controlling this interaction remained poorly understood. In this study, we used RSV and its small brown planthopper (SBPH) vector as a working model to elucidate the molecular mechanisms underlying the entrance of RSV virions into SBPH midgut cells for virus circulative and propagative transmission. We have determined that this non-enveloped tenuivirus uses its non-structural glycoprotein NSvc2 as a helper component to overcome the midgut barrier(s) for RSV replication and transmission. In the absence of this glycoprotein, purified RSV virions were unable to enter SBPH midgut cells. In the RSV-infected cells, this glycoprotein was processed into two mature proteins: an amino-terminal protein (NSvc2-N) and a carboxyl-terminal protein (NSvc2-C). Both NSvc2-N and NSvc2-C interact with RSV virions. Our results showed that the NSvc2-N could bind directly to the surface of midgut lumen via its N-glycosylation sites. Upon recognition, the midgut cells underwent endocytosis followed by compartmentalization of RSV virions and NSvc2 into early and then late endosomes. The NSvc2-C triggered cell membrane fusion via its highly conserved fusion loop motifs under the acidic condition inside the late endosomes, leading to the release of RSV virions from endosomes into cytosol. In summary, our results showed for the first time that a rice tenuivirus utilized its glycoprotein NSvc2 as a helper component to ensure a proper interaction between its virions and SBPH midgut cells for its circulative and propagative transmission.

## Author summary

Over 75% of the known plant viruses are insect transmitted. Understanding how plant viruses interact with their insect vectors during virus transmission is a key step towards the successful management of plant viruses worldwide. Several models for the direct or indirect virus–insect vector interactions have been proposed for the non-persistent or semi-persistent virus transmissions. However, the mechanisms controlling the interactions between viruses and their insect vector midgut barriers are poorly understood. In this study, we demonstrated that the circulative and propagative transmitted rice stripe virus (RSV) utilized its glycoprotein NSvc2 as a helper component to ensure a specific interaction between its virions and SBPH midgut cells to overcome the midgut barriers inside this vector. This is the first report of a viral helper component mediated mechanism for persistent-propagative virus transmission. Our new findings and working model should expand our knowledge on the molecular mechanism(s) controlling the interaction between virus and its insect vector during virus circulative and propagative transmission in nature.

## Introduction

Arthropod insects play critical roles in epidemics of numerous animal and plant viruses [1–3]. Based on the mode of transmission, plant viruses can be classified into non-persistent, semi-persistent or persistent transmitted viruses [4–6]. For non-persistent or semi-persistent transmissions, plant viruses are retained inside insect stylets from a few minutes to several hours or on insect foregut surface for a few hours to several days. Upon probing or feeding on a host plant, viruses are quickly injected into plant cells, together with insect saliva [7–9]. The persistent transmitted plant viruses (non-propagative or propagative) can enter insect vector bodies, and then circulate and/or replicate inside the vectors for several days to weeks [10]. These persistent transmitted viruses need to pass insect midgut barrier(s), dissemination barrier(s), and then salivary gland barrier(s) prior to be transmitted to new host plants [6, 11, 12]. Insect midgut is often considered to be one of the major barriers for successful persistent virus transmissions. During the process of passing through midgut barrier(s), proper interactions between viruses and vectors are needed. To date, the mechanism(s) controlling the interactions between viruses and their insect vector midgut barrier(s) are poorly understood.

Helper component-mediated mechanisms have been reported for the non-persistent, semi-persistent and persistent-nonpropagative transmitted plant viruses, respectively [4, 13–15]. For example, virions of non-persistent or semi-persistent transmitted plant viruses were reported to interact with the cuticular proteins inside the insect mouthpart or foregut [16, 17], and these virus–insect interactions required virally encoded non-structural helper factors as molecular bridges [13, 14]. Viruses in the genus *Potyvirus* are known to encode a helper component proteinase (HC-Pro) that can act as a molecular bridge for the interaction between potyvirus virions and its aphid vectors [18–20]. Members in the genus *Caulimovirus* encode a different helper factor that can help virions to retain on insect maxillary stylets [21–23]. Virions of multiple persistent (including propagative and non-propagative) transmitted plant viruses (e.g., luteovirus [24, 25], geminivirus [26, 27], reovirus [28, 29], tospovirus [30, 31], and plant rhabdovirus [32, 33]) were reported to bind directly to insect midgut cells, whereas these bindings depended on virions surface-exposed proteins. Faba bean necrotic yellows virus, a persistent-nonpropagative nanovirus, was found to require a helper factor for transmission by its aphid vector. To date, however, no persistent-propagative transmitted plant viruses were reported to rely on virally encoded helper proteins for their transmission.

Rice stripe virus (RSV) is transmitted by SBPH in a circulative and propagative manner, and often causes severe losses to rice production in China and many other countries in Asia [34, 35]. The genome sequence of plant-infecting tenuivirus is similar to the members of animal-infecting *Phlebovirus* in the order of *Bunyavirales*. Most members in the order *Bunyavirales* are known to produce membrane-enveloped spherical virions with two surface-exposed glycoproteins, and these glycoproteins are important for virus entrance into host cells or for vector transmission [31, 36, 37]. Virions of tenuiviruses are filamentous and do not have envelope membranes [38–40]. RSV also encodes a glycoprotein NSvc2 (92 kDa), which is further processed into an amino-terminal part protein known as NSvc2-N (40 kDa) and a carboxyl-terminal part protein known as NSvc2-C (50 kDa) [41, 42]. However, this glycoprotein is not present in the purified RSV virions [43, 44]. Based on the published reports, we hypothesized that RSV must use a different strategy to overcome the midgut barrier(s) for its insect transmission.

To validate this hypothesis, we conducted multiple experiments on the interaction between RSV and SBPH during virus entrance into insect vector midgut. We have now determined that this virus uses a viral glycoprotein NSvc2 as a helper component to overcome SBPH midgut barrier(s) for its persistent-propagative transmission. We have also determined that in the absence of NSvc2, RSV virions were unable to enter SBPH midgut cells. Our results further demonstrated that this glycoprotein acted as a critical helper component to ensure the proper interaction between RSV virions and SBPH midgut cells. Both NSvc2-N and NSvc2-C interacted with RSV virions and NSvc2-N bound directly to the midgut barrier(s). Upon successful interaction, the midgut cells underwent endocytosis followed by compartmentalization of RSV virions, NSvc2-N and NSvc2-C complexes (referred to RSV virions:NSvc2-N:NSvc2-C complex thereafter) inside the early and then late endosomes. NSvc2-C triggered membrane fusion under the acidic condition inside the late endosomes to release RSV virion:NSvc2-N complexes into the cytosol. These new findings expanded our knowledge on the interactions between virions and their insect vectors during plant virus persistent transmissions.

## Results

### Association of NSvc2 with RSV virions inside SBPH midgut

**Fig 1:**
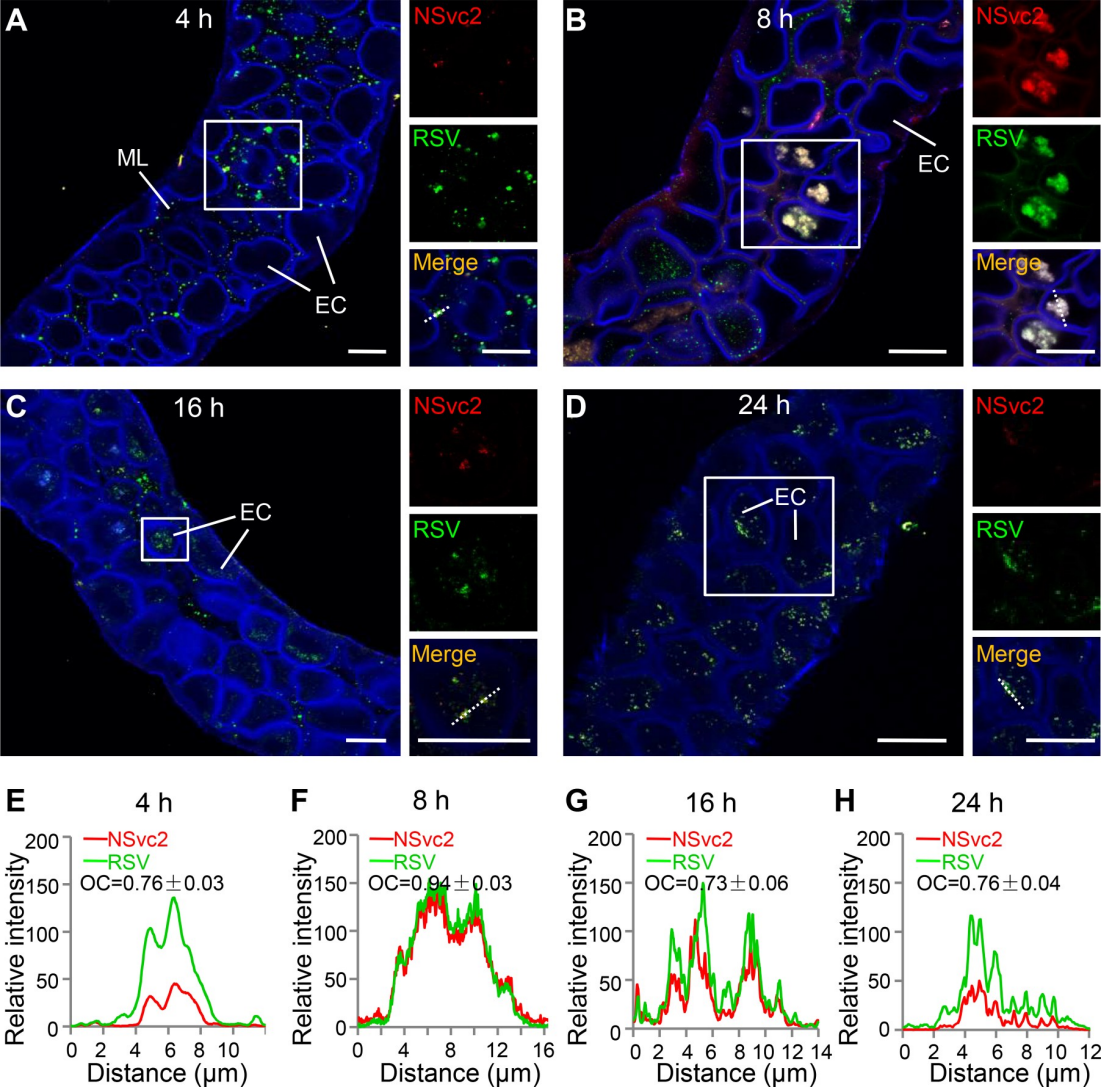
NSvc2 associated with RSV virions in SBPH midgut. (A) NSvc2 (red) and RSV virions (green) were co-localized in the midgut lumen and on the surface of intestinal microvillus (blue) at 4 h post feeding on the RSV-infected rice plants. The presence of RSV virions, NSvc2, and actin were detected with antibodies specific for RSV NP, NSvc2-N, or actin. The boxed region in each image was enlarged and shown in three different panels on the right side. The detection signal for NSvc2 is in red, the detection single for virions is in green, and the merged detection signals is in yellow. (B) Co-localization of NSvc2 and RSV virions in vesicle-like structures in epithelial cells at 8 h post feeding. (C) Co-localization of NSvc2 and RSV virions in the epithelial cells at 16 h post feeding. (D) NSvc2 and RSV virions complexes were detected in the epithelial cells of a midgut at 24 h post feeding. (E–H) Analyses of overlapped fluorescence spectra from NSvc2 (red) and RSV virions (green) at different stages. Fluorescence signals were from the white dashed line. The overlap coefficient (OC) values were determined using the LAS X software. ML, midgut lumen; EC, epithelial cells; Bar, 25 μm.

To examine whether NSvc2 plays important role(s) in RSV circulative-propagative transmission, we first conducted a time course study on the localizations of NSvc2 and RSV virions inside the midgut of SBPH during RSV acquisition. SBPHs were fed on RSV-infected rice seedlings and then collected at 4, 8, 16 and 24 h post feeding (30 SBPHs per time point), respectively. The collected insects were dissected and analyzed for the presence of NSvc2 and RSV virions by a double-immunolabeling using antibodies against RSV NSvc2-N or virion-surface nucleocapsid protein (NP). As shown in Fig 1A, RSV virions (green) had accumulated inside the midgut lumen at 4 h post feeding on the RSV-infected rice seedlings. In the same tissues, NSvc2 was also detected (red) and some of the NSvc2 signals were co-localized with RSV virions on the actin antibody-labelled intestinal microvillus (blue) (Fig 1A and S1A Fig), which are cellular membrane protrusions composed of dense bundles of cross-linked actin filaments at the surface of midgut epithelial cells [45]. The overlapped coefficient (OC) value for the red and green labeling signals was 0.76 ± 0.03 (Fig 1E), indicating that NSvc2 and RSV virions were localized close to each other. At 8 h post feeding, NSvc2 was found to co-localize with RSV virions in various sized vesicle-like structures inside the epithelial cells (Fig 1B and S1A Fig). Analysis of the OC value showed again that NSvc2 and RSV virions were localized close to each other (Fig 1F). At 16 h post feeding, RSV virions were detected together with NSvc2 in the cytosol of the midgut epithelial cells, with an OC value of 0.73 ± 0.06 (Fig 1C and 1G and S1A Fig). Even at 24 h post feeding, NSvc2 was still co-localized with RSV virions, with an OC value of 0.76 ± 0.04 (Fig 1D and 1H and S1A Fig). The SBPHs fed on the healthy rice seedlings did not give positive signals for NSvc2 (red) and RSV virions (green) (S2A and S2B Fig). These data indicated that the RSV-encoded NSvc2 was associated with RSV virions inside SBPH midgut during insect feeding on RSV-infected rice plants.

### NSvc2 is required for RSV virion entrance into SBPH midgut cells

**Fig 2:**
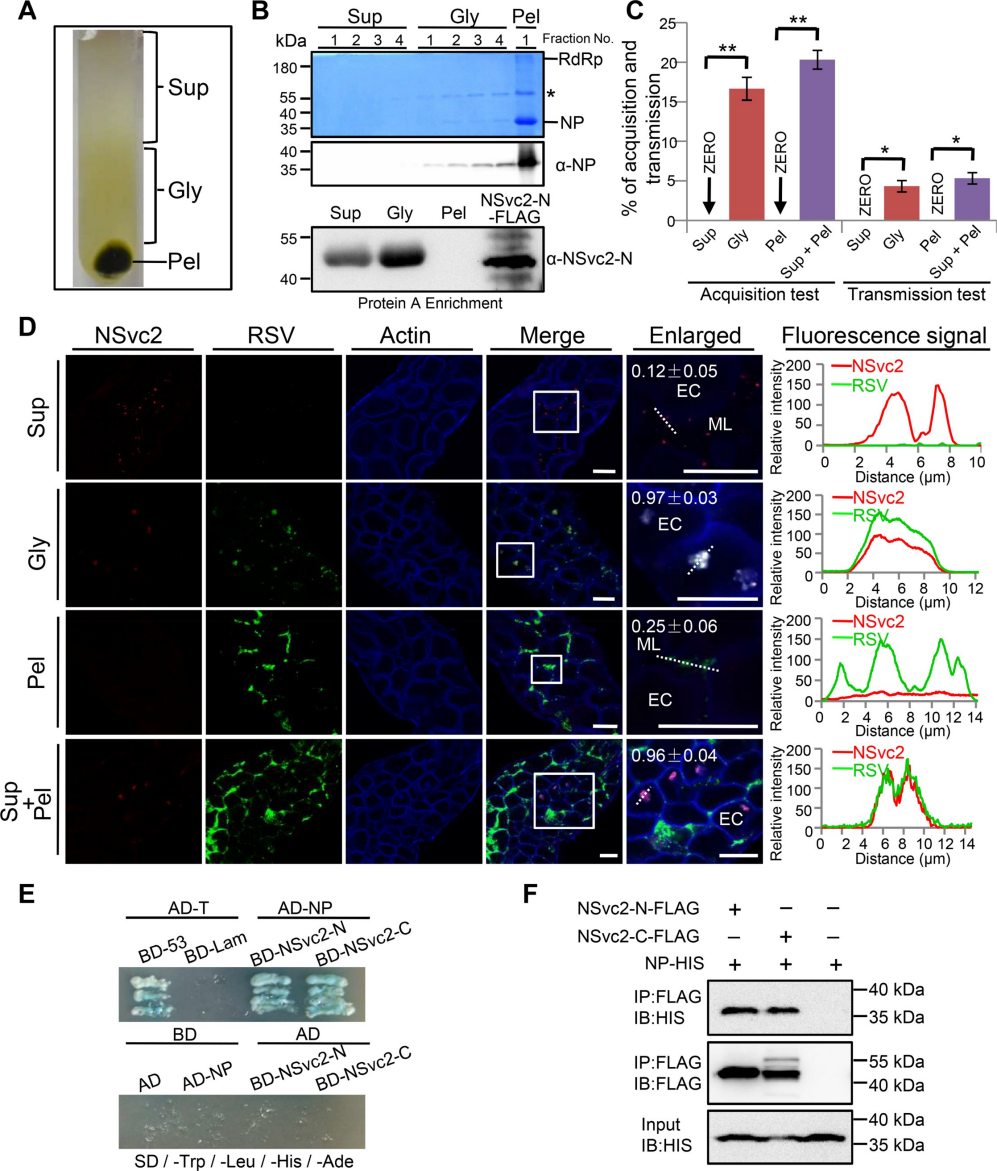
NSvc2 protein is required for the entrance of RSV virions into SBPH midgut. (A) NSvc2 is absent in purified RSV virions. Extract from RSV-infected rice plants was loaded on the top of a 4 mL 20% glycerol cushion. The supernatant fractions (Sup 1 to 4), glycerol fractions (Gly 1 to 4) and the pellet (Pel) were collected separately after ultra-centrifugation. (B) The collected samples were analyzed in the SDS-PAGE gels followed by Coomassie blue staining or by immunoblotting using antibodies specific for RSV NP or NSvc2-N. NSvc2-N was enriched using protein A beads. Sizes of the protein bands are shown on the left. Asterisk indicates an RSV NP dimer band. (C) Statistic analysis of RSV acquisition rate and transmission rate by SBPHs after feeding on different fractions. Each bar represents three independent biological repeats from each experiment. *, p < 0.05 and **, p < 0.01 by the student t-test. (D) Immunofluorescence labeling of NSvc2 (red) and RSV virions (green) in the midguts of the SBPHs fed with the combined supernatant fractions, combined glycerol fractions, the resuspended pellet sample or the mixture of the combined supernatant fractions and the resuspended pellet sample. The boxed regions are enlarged and shown on the right side of the merged images. Overlapping fluorescence spectra analyses were done for the white dashed line indicated areas shown in the right panels. The overlap coefficient (OC) values were determined using the LAS X software. ML, midgut lumen; EC, epithelial cells; Bar, 25 μm. (E) Yeast two-hybrid assay for the interaction between RSV NP and NSvc2-N, or between RSV NP and NSvc2-C. RSV NP was fused to a GAL4 activation domain (AD-NP), and NSvc2-N or NSvc2-C was fused to a GAL4 binding domain (BD-NSvc2-N, BD-NSvc2-C). Yeast cells were co-transformed with the indicated plasmids and were assayed for protein-protein interactions on the synthetic dextrose -Trp/-Leu/-His/-Ade medium. Co-transformed the plasmids AD-T and BD-53 were used as a positive control while co-transformed AD-T and BD-Lam were used as negative controls. (F) Co-immunoprecipitation assay for the interaction between NSvc2-N and NP, or between NSvc2-C and NP. NSvc2-N or NSvc2-C was fused to a FLAG tag and RSV NP was fused to a 6x HIS tag. Individual recombinant proteins were expressed in Sf9 cells followed by the co-immunoprecipitation assays using a FLAG tag specific or a HIS tag specific antibody. IB:FLAG, immunoblot with a FLAG tag specific antibody; IB:HIS, immunoblot with a HIS tag specific antibody; IP:FLAG, immunoprecipitation with a FLAG tag specific antibody. The sizes of the protein bands are shown on the right side.

**Fig 3:**
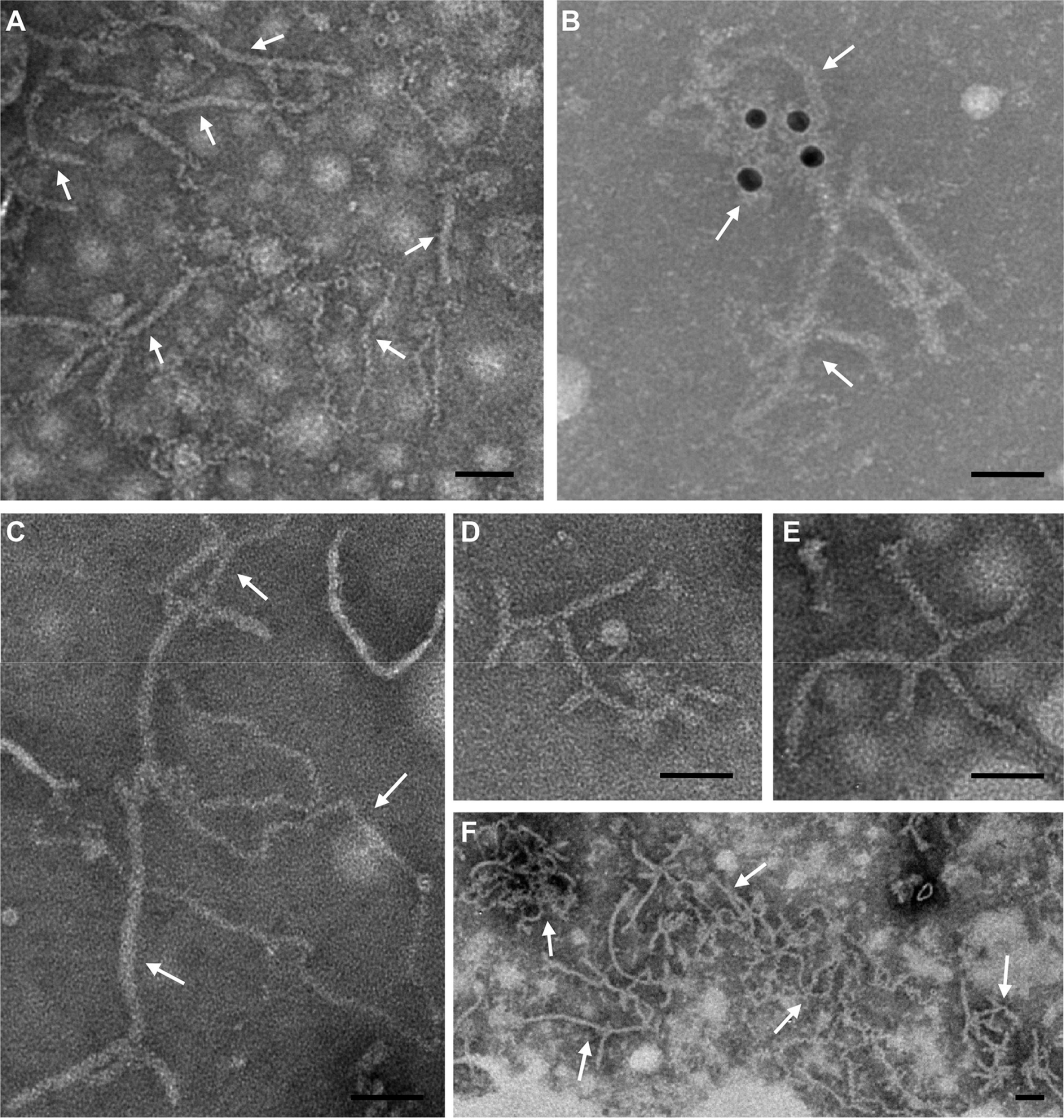
Electron Microscopy and immunogold labelling of purified RSV virions. (A) An electron microscopic image of RSV virions from the resuspended pellet sample. (B) Immunogold labeling of purified RSV virions using an RSV NP specific antibody followed by a goat anti-mouse IgG conjugated with 15 nm gold particles. (C) A negative control of immunogold labeling assay using only the goat anti-mouse IgG conjugated with 15 nm gold particles. (D-F) Immunogold labeling of purified RSV virions with an RSV NSvc2-N specific antibody followed by a goat anti-rabbit IgG conjugated with 8 nm gold particles. Arrows indicate filamentous RSV virions. Bar, 50 nm.

RSV virions were purified from RSV-infected rice seedlings through ultracentrifugation using a 20% glycerol cushion. After ultracentrifugation, four different supernatant fractions starting from the top (Sup1 to Sup4), four 20% glycerol phase fractions starting from the top (Gly1 to Gly4), and the pellet (Pel) were collected and analyzed individually by immunoblotting assays using an antibody against RSV NP (35 kDa) or NSvc2-N (Fig 2A and 2B). Results showed that the resuspended pellet sample contained RSV virions, and the four supernatant fractions (Sup1 to Sup4) contained NSvc2 protein only. In contrast, the four glycerol phase fractions (Gly1 to Gly4) contained both RSV virions and NSvc2 (Fig 2B). Transmission Electron Microscopy showed that numerous filamentous RSV virions were present in the resuspended pellet sample (Fig 3A). Immunogold labeling using a gold-labeled NP specific antibody showed that the immunogold particles were attached to the purified RSV virions (Fig 3B). When a NSvc2 specific antibody was used in the immunogold labeling assay, no immunogold particle was observed together with the purified RSV virions (Fig 3D to 3F), confirming that the purified RSV virions do not contain NSvc2.

To further verify the above findings, SBPHs were allowed to feed on a mixture of sucrose and the combined supernatant fractions, sucrose and the combined glycerol fractions, sucrose and the resuspended pellet sample, sucrose alone, or the combined supernatant fractions and the resuspended pellet samples through two layers of stretched parafilm for 24 h. As shown in Fig 2D (row 2), NSvc2 (red) and RSV virions (green) were detected together inside the epithelial cells of SBPHs fed on the mixture containing sucrose and the combined glycerol fractions. NSvc2 was, but not RSV virions, detected inside the midgut lumen of the SBPHs fed on the mixture containing sucrose and the combined supernatant fractions (Fig 2D, row 1). RSV virions were detected inside the midgut lumen but not inside the epithelial cells of the SBPHs fed on the mixture containing sucrose and the resuspended pellet samples (Fig 2D, row 3). When insects fed on the mixture containing sucrose, the combined supernatant fractions and the resuspended pellet samples, however, both NSvc2 and RSV virions were detected inside the epithelial cells (OC value = 0.96 ± 0.04; Fig 2D, row 4), similar to that found for the SBPHs fed on the mixture containing sucrose and the combined glycerol phase fractions (OC value = 0.97 ± 0.03). No NSvc2 or RSV virions was detected inside the midgut of the SBPHs fed on the mixture containing sucrose and the combined glycerol phase fractions from the healthy rice seedlings (S2C Fig).

To further investigate the roles of NSvc2 in RSV acquisition and transmission by SBPH, SBPHs were fed with a mixture containing sucrose and the combined supernatant fractions, a mixture containing sucrose and the combined glycerol phase fractions, a mixture containing sucrose and the resuspended pellet sample, or a mixture containing sucrose, the mixed supernatant fractions and the resuspended pellet sample for 48 h and then on rice seedlings. The rate of SBPH acquired RSV and the rate of SBPH transmitted RSV to rice seedlings were determined by immuno-dot blot assays. The results showed that after feeding on the mixture containing sucrose, the combined supernatant fractions and the resuspended pellet samples, RSV virions were successfully transmitted to rice seedlings (Fig 2C and S1 Table). No RSV acquisition and transmission were observed for the SBPHs fed on sucrose only or on the mixture containing sucrose and the resuspended pellet sample.

We then performed yeast two-hybrid and co-immunoprecipitation assays. Our results showed that both NSvc2-N and NSvc2-C interacted with RSV NP (Fig 2E and 2F), suggesting that RSV NSvc2 played a critical role in mediating the entrance of RSV virions into SBPH midgut cells.

### Recombinant soluble NSvc2-N binds directly to midgut cells and inhibits subsequent RSV acquisition by SBPH

A previous research has shown that NSvc2 can be further processed into two mature glycoproteins, namely NSvc2-N and NSvc2-C [42]. S3A Fig illustrated the predicted positions of the signal peptide (SP), transmembrane (TM) regions and glycan sites within NSvc2. To investigate the roles of NSvc2-N in RSV transmission, we deleted the TM region and expressed the soluble part of NSvc2-N protein (referenced to as NSvc2-N:S thereafter, S3B Fig) in *Spodoptera frugiperda* (Sf9) cells using a recombinant baculovirus expression system. After purification using the Ni-NTA agarose, the expression of the recombinant NSvc2-N:S was confirmed by Western blot assays using an anti-NSvc2-N polyclonal antibody (S3C Fig).

**Fig 4:**
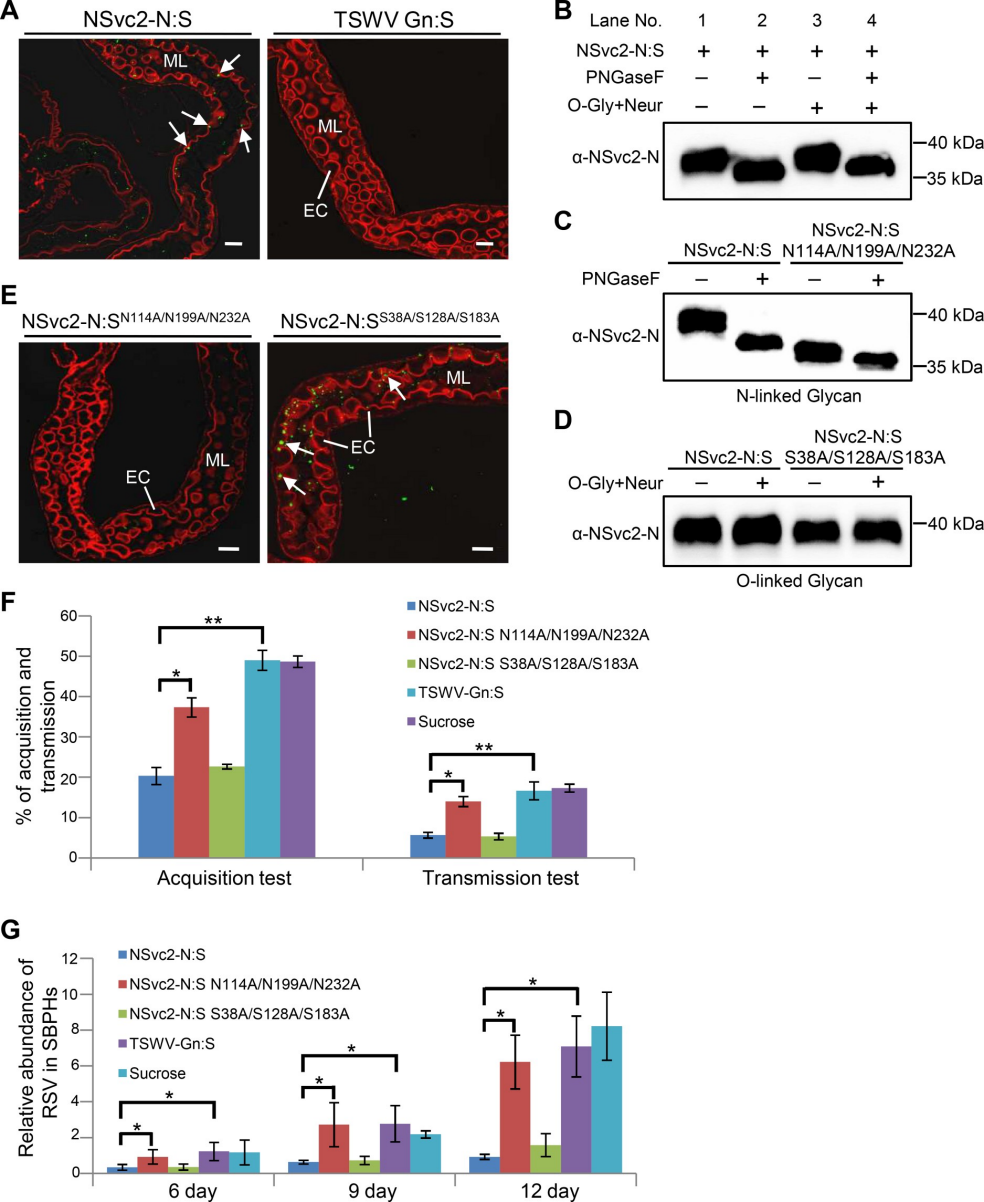
N-glycosylation of NSvc2-N is essential for the recognition of midgut surface receptors. (A) Binding of purified NSvc2-N:S to microvillus surface in SBPH midgut. Arrows indicate the accumulation of NSvc2-N:S (green, left panel) in the midgut lumen. Purified TSWV Gn:S was used as a negative control in this study (right panel). ML, midgut lumen; EC, epithelial cells; Bar, 25 μm. (B) Enzymatic de-glycosylation of NSvc2-N:S. Purified NSvc2-N:S was incubated with PNGaseF or O-Glycosidase + Neuraminidase (O-Gly + Neur) to determine the types of glycans. PNGaseF was used to remove the N-linked glycans, and O-Gly + Neur were used to remove the O-linked glycans. (C) The NSvc2-N:SN114A/N199A/N232A mutant was treated with PNGaseF. (D) The NSvc2-N:SS38A/S128A/S183A mutant was treated with O-Gly + Neur. (E) The NSvc2-N:SN114A/N199A/N232A mutant failed to bind SBPH midgut (left) but the O-glycosylated NSvc2-N:SS38A/S128A/S183A mutant did (right). ML, midgut lumen; EC, epithelial cells; Bar, 25 μm. (F) Percentages of RSV acquisition and transmission by SBPHs pre-fed with NSvc2-N:S, NSvc2-N:SN114A/N199A/N232A mutant, NSvc2-N:SS38A/S128A/S183A mutant, TSWV Gn:S, or sucrose only. The experiment was performed three times with 100 SBPHs per treatment. *, p < 0.05 and ** p < 0.01 by the student t-test. (G) Quantitative RT-PCR analysis of RSV acquisition by SBPHs at 6, 9- or 12-days post RSV feeding. The SBPHs were pre-fed with NSvc2-N:S, NSvc2-N:SN114A/N199A/N232A mutant, NSvc2-N:SS38A/S128A/S183A mutant, TSWV Gn:S, or sucrose only for 24 h, then were allowed to feed on the RSV-infected rice plants for 48 h. The experiment was performed three times. *, p < 0.05 by the student t-test.

To confirm that NSvc2-N:S can bind midgut epidermal microvillus, SBPHs were allowed to feed on purified NSvc2-N:S for 3 h followed by a 12 h feeding on a sucrose solution to remove unbound NSvc2-N:S. Results of immuno-labeling assays showed that NSvc2-N:S (green) could be readily detected in the midgut lumen near the surface of epithelial cells along the alimentary canal (Fig 4A). As a negative control, SBPHs were allowed to feed on the *Tomato spotted wilt virus* (TSWV)-encoded soluble glycoprotein (Gn:S), known to bind thrip midgut cells [30]. As expected, the TSWV Gn:S (green) was not detected inside the SBPH midgut (Fig 4A).

Based on the above findings, we further hypothesized that pre-acquisition of NSvc2-N:S could prevent RSV infection through blocking the RSV specific receptors on the midgut. To test this hypothesis, SBPHs were allowed to feed on the purified NSvc2-N:S for 24 h and then on the RSV-infected rice plants for 48 h. The midguts were dissected from the SBPHs and probed with the RSV NP or NSvc2-N specific antibody. Under the confocal microscope, the labeled RSV virions were found inside the midgut lumen, while the virions in the midgut epithelial cells were significantly reduced in the SBPHs pre-fed with purified NSvc2-N:S (S4A and S4D Fig). In contrast, RSV virions were detected in the midgut epithelial cells of the SBPHs pre-fed with TSWV Gn:S or with sucrose alone (S4B–S4D Fig).

To further elucidate the roles of NSvc2-N, SBPHs pre-fed with NSvc2-N:S were allowed to feed on the RSV-infected rice plants for 48 h followed by examinations of RSV acquisition and transmission. Immuno-dot blot assays showed that pre-feeding SBPHs with NSvc2-N:S significantly reduced the rate of RSV acquisition and transmission to rice seedlings compared with that in the SBPHs pre-fed with TSWV Gn:S or with sucrose only (Fig 4F and S2 Table). We then performed quantitative RT-PCR to determine the relative levels of RSV RNA in the assayed SBPHs at different time points. The results showed that RSV accumulation in the SBPHs pre-fed with NSvc2-N:S was significantly reduced compared with that in the SBPHs pre-fed with TSWV-Gn:S or with sucrose alone (Fig 4G). These findings indicated that NSvc2-N:S could inhibit RSV entrance into SBPH midgut cells.

### N-glycosylation of NSvc2-N is required for the entry of RSV into SBPH midgut(s)

Computer-assisted modeling suggested that NSvc2 might be modified through glycosylation (S3A Fig). To confirm this prediction, purified NSvc2-N:S was incubated with PNGaseF (an N-glycosidase) to remove the N-linked glycan or with O-Glycosidases and Neuraminidase (O-Gly + Neur) to remove the O-linked glycan. Subsequent SDS-PAGE and immunoblotting assays showed that the purified NSvc2-N:S protein band was shifted in the gel after the PNGaseF treatment compared with the non-treated NSvc2-N:S (Fig 4B, compare lane 1 with lane 2). No clear protein band shift was observed after the treatment with O-Gly + Neur (Fig 4B, compare lane 1 with lane 3). When NSvc2-N:S was treated with PNGaseF and O-Gly + Neur, the protein band shifted as that shown by the PNGaseF-treated NSvc2-N:S (Fig 4B, compare lane 2 with lane 4), indicating that the NSvc2-N contains the N-linked glycan.

We then introduced alanine-substitution mutations at the putative N-linked glycan sites of NSvc2-N:S to produce NSvc2-N:S^N114A/N199A/N232A^ or at the putative O-linked glycan sites of NSvc2-N:S to produce NSvc2-N:S^S38A/S128A/S183A^. These two mutants were purified as describe above for NSvc2-N:S followed by the enzymatic deglycosylation treatments. Results showed that, without PNGaseF treatment, mutant NSvc2-N:S^N114A/N199A/N232A^ showed a protein band shift similar to that shown by the PNGaseF-treated NSvc2-N:S (Fig 4C, compare lane 2 with lane 3). With PNGaseF treatment, the band position of NSvc2-N:S^N114A/N199A/N232A^ mutant further shifted (Fig 4C, compare lane 3 with lane 4). In this study, no O-linked glycan modification was detected for mutant NSvc2-N:S^S38A/S128A/S183A^ (Fig 4D). These results indicated that among the three residues (N114, N199 and N232 in NSvc2-N:S), one or more residues were indeed N-glycosylated.

To investigate whether the N-linked glycosylation can affect the entrance of RSV into midgut(s), SBPHs were fed with the two mutant proteins, respectively, for 3 h followed by a 12 h feeding on a sucrose solution to clean insect alimentary canals. The SBPHs fed with the NSvc2-N:S^N114A/N199A/N232A^ mutant showed almost no labeling signal at the surface of midgut microvillus (Fig 4E, left). In contrast, the SBPHs fed with the NSvc2-N:S^S38A/S128A/S183A^ mutant showed labeling signal (Fig 4E, right). To determine whether the effect of NSvc2-N on RSV acquisition and transmission could be affected by the mutation caused by N-glycosylation, we pre-fed SBPHs with the NSvc2-N:S^N114A/N199A/N232A^ mutant and then tested RSV acquisition and transmission through immuno-dot blot assays. The results showed that the SBPHs pre-fed with this mutant protein gave similar RSV acquisition and transmission rates as the SBPHs pre-fed with sucrose only (Fig 4F and S2 Table). Our quantitative RT-PCR results showed that the accumulation level of RSV RNA in the SBPHs pre-fed with the NSvc2-N:S^N114A/N199A/N232A^ mutant was similar to that in the SBPHs pre-fed with TSWV-Gn:S or with sucrose only (Fig 4G). Consequently, we conclude that modification of NSvc2-N through N-glycosylation is important for RSV accumulation in SBPHs.

### Entrance of RSV virions into and then release from endosomes

**Fig 5:**
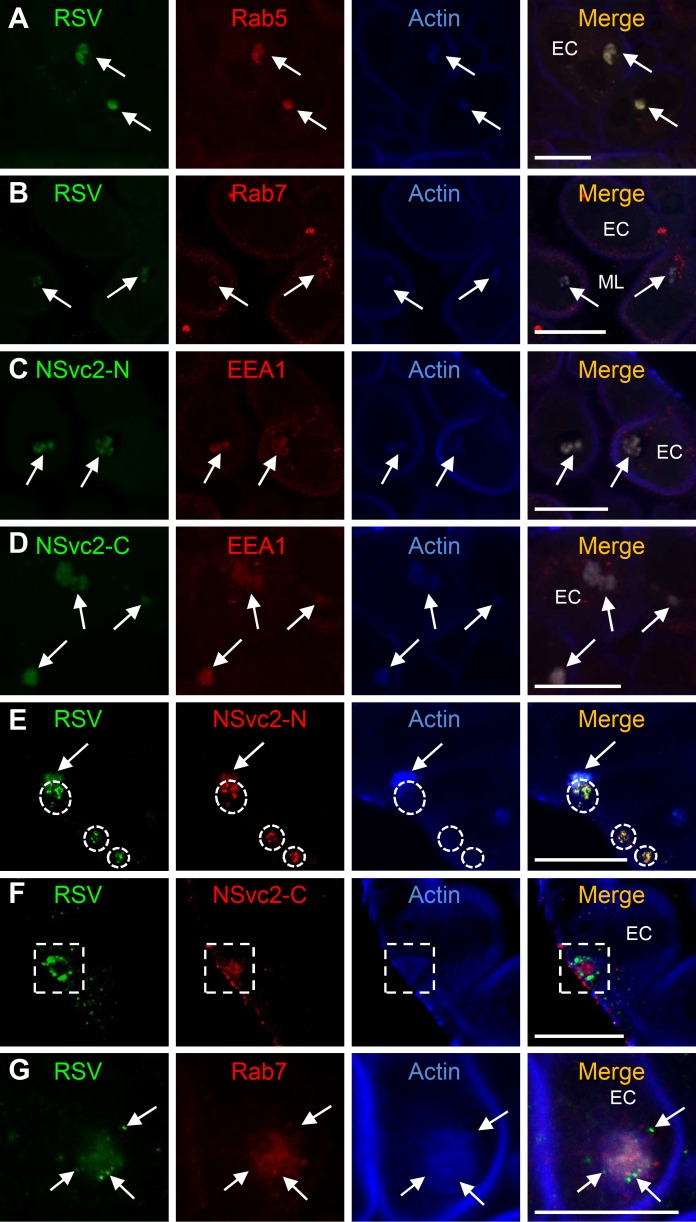
Localizations of RSV virions, NSvc2-N and NSvc2-C in SBPH midgut epithelial cells. (A and B) RSV virions were detected in the early endosomes inside midgut epithelial cells by labeling using the Rab5 antibody (A) or in the late endosomes by labeling using the Rab7 antibody (B). Localization of RSV virions in the Rab5 antibody-labeled early endosomes or in the Rab7 antibody-labeled late endosomes are indicated with arrows. Actin antibody was used to visualize actin filaments inside the midgut epithelial cells. Bar, 25 μm. (C and D) NSvc2-N and NSvc2-C were detected inside the early endosomes labeled with the EEA1 antibody. Co-localizations of NSvc2-N or NSvc2-C with EEA1 or actin in different endosomes are indicated with arrows. Bar, 25 μm. (E) RSV virions:NSvc2-N complexes were released from the actin labelled late endosomes into the cytosol of epithelial cells. The white dashed cycles indicate the regions where the RSV virions:NSvc2-N complexes were detected in the cytosol. Bar, 25 μm. (F) RSV virions was released into the cytosol of epithelial cells but not NSvc2-C. The white dashed boxes indicate a region where RSV virions were released from the endosomes while NSvc2-C was still associated with endosomes. Bar, 25 μm. (G) RSV virions were released from the Rab7 antibody-labeled late endosomes into the cytosol of epithelial cells. The released RSV virions are indicated with arrows. ML, midgut lumen; EC, epithelial cells; Bar, 25μm.

Endocytosis is an important process during the entrance of several circulative-transmitted animal-infecting viruses into animal cells [46]. Because the above results had indicated that RSV virions and NSvc2-N accumulated together inside vesicles-like structures in SBPH midgut cells (Fig 1B), we decided to investigate whether these vesicles were endosomes using early or late endosome specific markers (e.g., Rab5, EEA1 and Rab7) as previously reported [47, 48]. Results of this study showed that the RSV virion labeling signal was indeed co-localized with the labeling signal from the early endosome Rab5 marker or from the late endosome Rab7 marker (Fig 5A and 5B). In the same study, the labeling signal from NSvc2-N or NSvc2-C was co-localized with the labeling signal from the early endosome EEA1 marker (Fig 5C and 5D).

To examine the potential role(s) of NSvc2-C, SBPHs were allowed to feed on RSV-infected rice seedlings for 4, 8, 16 or 24 h, and then tested by immunofluorescence labeling assays. NSvc2-C showing red labeling signal was observed together with the labeling signal from RSV virions (green) at the surface of microvillus (blue) (S5A and S5E Fig, OC value = 0.86 ± 0.04), and in the endosomal-like vesicles inside epithelial cells (S5B and S5F Fig, OC value = 0.95 ± 0.03) at 4 and 8 h post feeding. At 16 and 24 h post feeding, the labeling signal from RSV virions (green) was observed alone in the cytosol of the epithelial cells (S5C and S5G Fig, OC value = 0.18 ± 0.02; S5D and S5H Fig, OC value = 0.32 ± 0.06), suggesting that NSvc2-C was retained inside the endosomal-like vesicles while RSV virions were released from the vesicles into the cytosol. To confirm this finding, we double-immunolabeled RSV virions and NSvc2-N or RSV virions and NSvc2-C inside the cells and monitored the changes of the localization patterns in the endosomes starting from 16 to 24 h post feeding. The results showed that both RSV virions and NSvc2-N were released from the endosomes and stayed together in the cytosol (Fig 5E, white dashed circles; Fig 5G, arrows). In contrast, NSvc2-C stayed inside the endosomes and thus was not found in the cytosol of the epithelial cells (Fig 5F, white dashed box). This finding indicated that RSV virions, NSvc2-N and NSvc2-C formed complexes and entered into the endosomes, and later the RSV virions:NSvc2-N complexes were released from the endosomes into the cytosol.

### NSvc2-C induces insect cell membrane fusion under acidic condition

**Fig 6:**
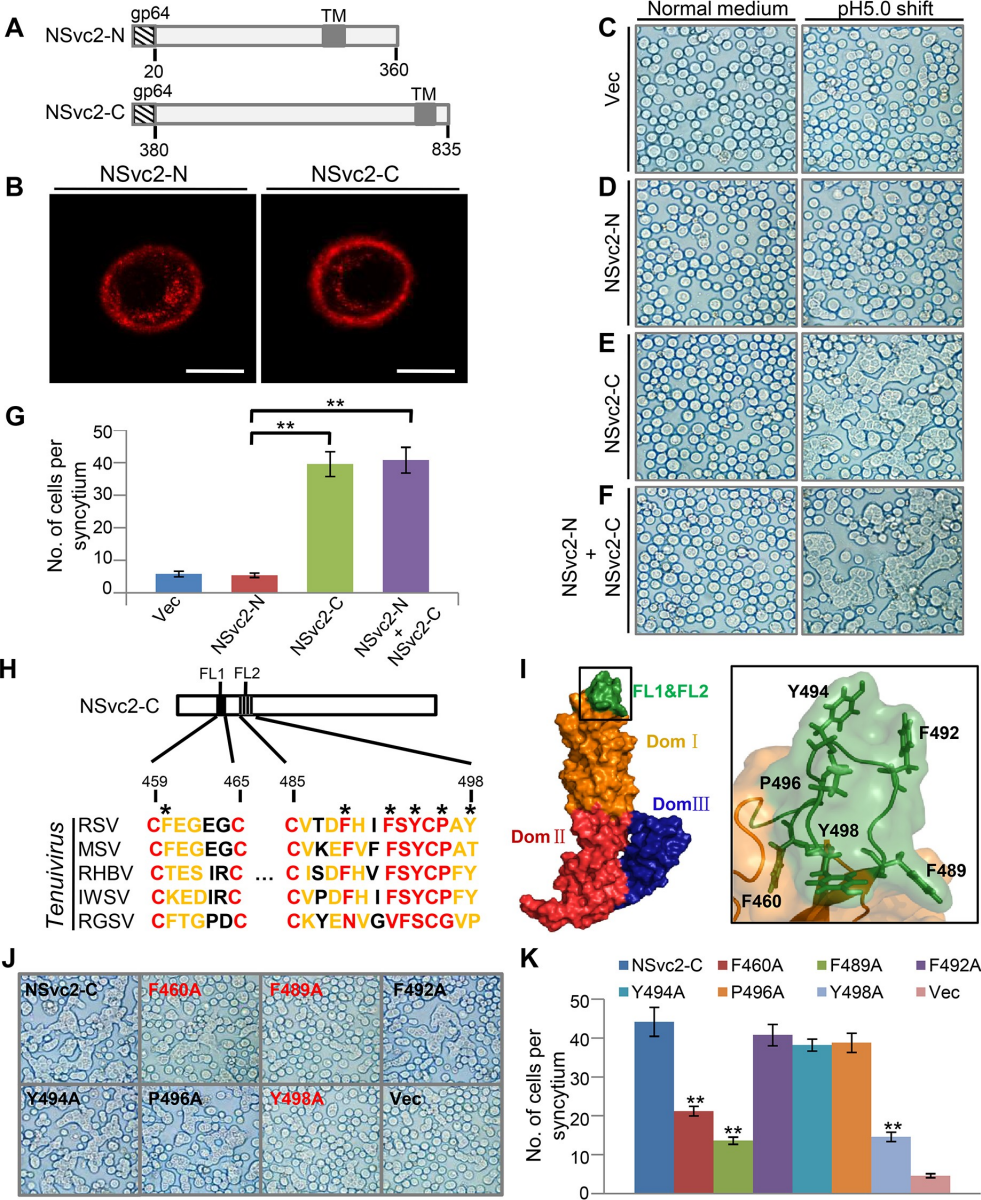
NSvc2-C hydrophobic fusion-loop motifs are required for cell-cell membrane fusion. (A) Schematic diagrams showing recombinant NSvc2-N and NSvc2-C. Baculovirus gp64 signal peptide was used to replace the original signal peptide at the N-terminus of NSvc2-N or NSvc2-C. (B) Immunofluorescence labeling of the recombinant NSvc2-N or NSvc2-C in Sf9 cells. The Sf9 cells were infected with baculoviruses expressing the recombinant NSvc2-N or NSvc2-C (MOI = 5). The infected cells were probed with the NSvc2-N or NSvc2-C specific antibody at 48 h post infection followed by a TRITC fluorescence-labeled secondary antibody. Bar, 10 μm. (C–F) Fusogenic activity assays using Sf9 cells expressing the recombinant NSvc2-N, NSvc2-C or both (NSvc2-N + NSvc2-C). The Sf9 cells were infected with individual recombinant baculoviruses (MOI = 5). At 48 h post transfection, the growth medium was replaced with a PBS, pH 5.0, for 2 min and then changed back to the normal growth medium for 4 h. Formation of syncytium was observed under a microscope. (G) The number of cells per syncytium was counted under the inverted microscope. The experiment was repeated three times. **, p < 0.01 by the student t-test. (H) Sequence alignment using the two fusion loop sequences (FL1 and FL2) from the NSvc2-C of five different tenuiviruses [RSV (NC_003754.1), maize stripe virus (MSV; U53224.1), rice hoja blanca virus (RHBV; L54073.1), Iranian wheat stripe virus (IWSV; AY312434.1) and rice grassy stunt virus (RGSV; KF438676.1)]. Red residues are highly conserved and yellow residues are semi-conserved. Residues indicated with asterisks are hydrophobic residues and were selected for site-directed mutagenesis. The resulting mutants were tested for their abilities to induce cell-cell membrane fusion. (I) Two fusion loops (FL1 and FL2, green) were found at the top of the 3D-structure model of NSvc2-C. Three different domains are shown in three different colors, and the boxed region of the 3D-structure is enlarged and shown on the right side. Locations of the hydrophobic residues are shown. (J and K) Analyses of fusogenic activities caused by the WT or mutant NSvc2-C. Sf9 cells were infected with the recombinant baculoviruses expressing the WT or mutant NSvc2-C for membrane fusion assays (J). The number of cells per syncytium was counted and analyzed (K). The experiment was repeated three times. **, p < 0.01 by the student t-test. Fig 6C–6F and 6J are bright field light microscopic images.

From early endosomes to late endosomes, the pH value inside the endosome lumen shifted from 6.5 to 5.0 [46]. The acidic condition inside the late endosomes could alter the conformations of virus proteins and trigger cell membrane fusion. To determine whether NSvc2-N and/or NSvc2-C played roles in cell membrane fusion under the acidic condition, we fused a signal peptide from a baculovirus protein (gp64) to NSvc2-N and NSvc2-C and expressed these two proteins individually in Sf9 cells through the recombinant baculovirus expression system (Fig 6A). We first confirmed the expressions of these two proteins by Western blot assays (S6C Fig). We then conducted immune-labeling assays to further confirm the above results. Under the laser scanning confocal microscope, red labeling signal representing NSvc2-N or NSvc2-C was observed in the Sf9 cell membranes (Fig 6B). We then tested NSvc2-N and NSvc2-C individually to see if they could trigger cell membrane fusion under an acidic condition. The Sf9 cells were first transfected with a baculovirus expressing NSvc2-N or NSvc2-C, and then treated with a PBS solution, pH 5.0, for 2 min followed by growth in a normal medium. At 4 h post acidic PBS treatment, numerous cell-cell fusions (syncytia) were observed for the Sf9 cells transfected with the baculovirus expressing NSvc2-C (Fig 6E). In contrast, no significant cell-cell fusion was observed for the Sf9 cells transfected with the baculovirus expressing NSvc2-N or with the empty baculovirus (Fig 6C and 6D). In addition, cell-cell membrane fusion was observed for the Sf9 cells co-infected with the baculovirus expressing NSvc2-N and the virus expressing NSvc2-C (Fig 6F and 6G), confirming that only RSV NSvc2-C played a role in cell-cell membrane fusion under the acidic condition.

### Conserved hydrophobic fusion-loop motifs in NSvc2-C are responsible for the fusogenic activity

To further investigate the function of NSvc2-C in cell-cell membrane fusion, we generated a three-dimensional (3D) model structure of NSvc2-C through a homology-based modeling approach (S6A and S6B Fig). The 3D model of NSvc2-C consisted of three distinct domains: domain I (yellow), II (red), and III (blue). Two putative fusion loops (green) were predicted at the top of the NSvc2-C model (S6B Fig). Similar loops were also reported to be responsible for cell-cell membrane fusion during animal virus infections [49, 50]. We then constructed three NSvc2-C mutants with one or two fusion loops (FL) deleted (i.e., ΔFL1, ΔFL2 and ΔFL1+ΔFL2). These mutants were infected individually into Sf9 cells and their expressions were confirmed individually by immunoblotting assays (S6D and S6E Fig). Fusogenic activities of the three deletion mutants were then examined in the Sf9 cells using the recombinant baculovirus expression system. Results showed that the number of syncytial cells induced by the ΔFL1 or ΔFL2 mutant was much less than that induced by the WT NSvc2-C. When the ΔFL1 + ΔFL2 mutant was expressed in the Sf9 cells, the number of syncytial cells was further decreased (S6F and S6G Fig). Based on this finding, we concluded that both fusion loops of NSvc2-C played important roles in cell-cell membrane fusion.

To identify the amino acid residue(s) important for the fusogenic activity, we aligned the RSV fusion loop sequences (Cys459-Cys465 [Loop 1] and Cys485-Tyr498 [Loop 2]) with the loop sequences from other four tenuiviruses (Fig 6H). The alignment result indicated that the two fusion loop sequences were relatively conserved among the five tenuiviruses. Six conserved hydrophobic amino acid residues (Phe460, Phe489, Phe492, Tyr494, Pro496 and Tyr498) were found in a unique vertex in the modeled NSvc2-C 3D structure (Fig 6I). Introduction of mutations into each of the six amino acid residues showed that three of them (F460A, F489A, and Y498A) were important for the membrane fusion activity (Fig 6J and 6K).

### Membrane fusion mutant of NSvc2-C is defective in releasing RSV virions from endosomes

**Fig 7:**
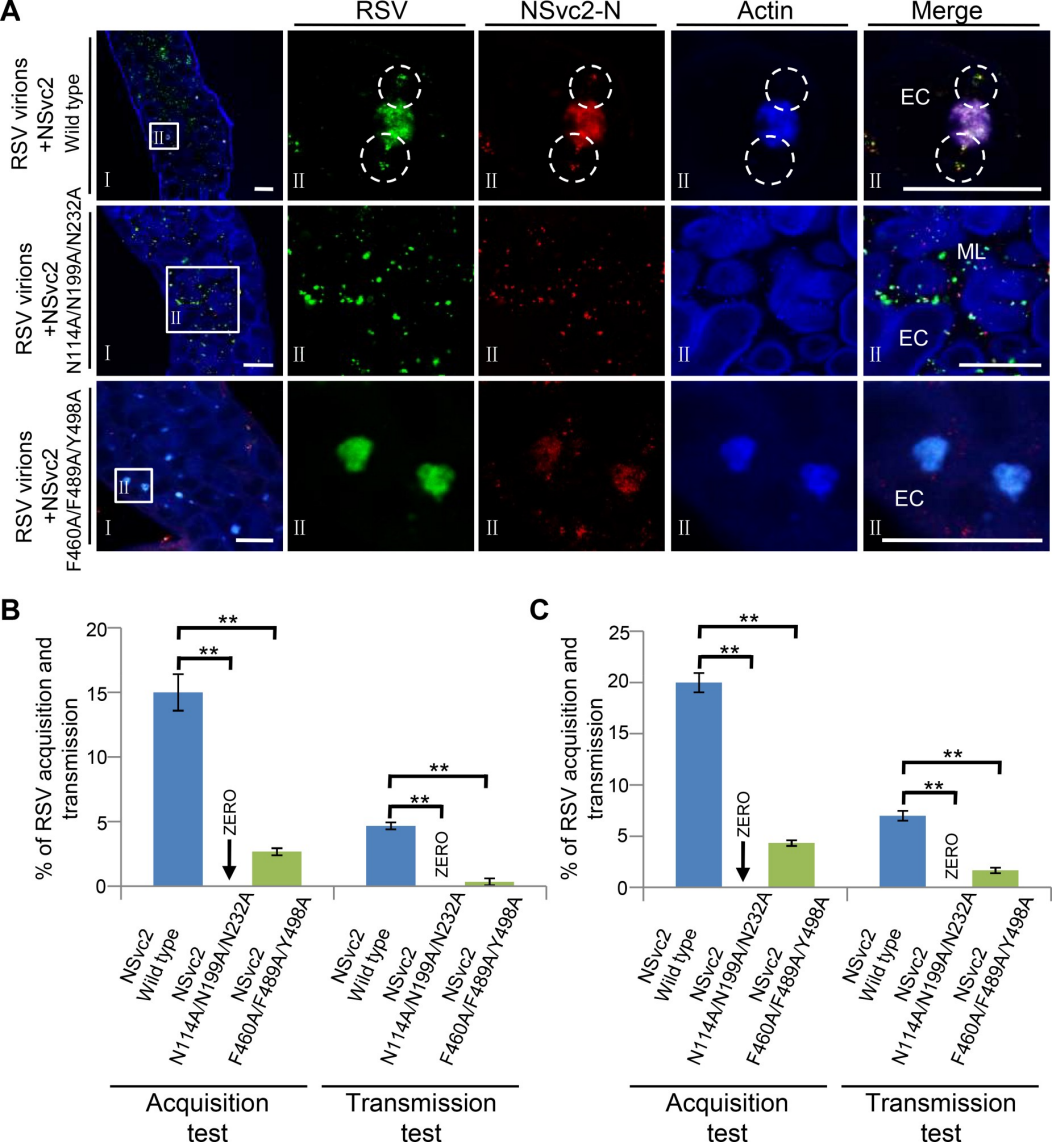
NSvc2 functioned as a helper factor to mediate the entrance of RSV virions into SBPH midgut cells. (A) Crude extract of Sf9 cells expressing NSvc2, NSvc2N114A/N199A/N232A or NSvc2F460A/F489A/Y498A was incubated with purified RSV virions for 3 h at 4°C, and then used to feed SBPHs for 24 h. Insect midguts were dissected and detected for the presence of RSV virions and NSvc2-N using specific antibodies. The boxed regions inside the left column images were enlarged and shown in the second to fifth columns on the right. The white circled areas show the release of RSV virions (green) and NSvc2-N (red) from endosomes into the cytosol. The actin labeling signal is shown in blue. ML, midgut lumen; EC, epithelial cells; Bar, 25 μm. (B) Percentages of SBPHs acquired or transmitted RSV. SBPHs were fed with the mixtures containing purified RSV virions and NSvc2, purified RSV virions and NSvc2N114A/N199A/N232A mutant or purified RSV virions and NSvc2F460A/F489A/Y498A mutant prior to virus transmission. (C) Percentages of SBPHs acquired or transmitted RSV. SBPHs were first fed with NSvc2, NSvc2N114A/N199A/N232A mutant or NSvc2F460A/F489A/Y498A mutant, and then fed with purified RSV virions prior to virus transmission. **, p < 0.01 by the student t-test.

The above results showed that RSV NSvc2-N and NSvc2-C had different functions during RSV acquisition by SBPH. To elucidate the role of NSvc2-C in RSV entrance into midgut cells during virus acquisition, we developed a new system to analyze the wild type NSvc2 (NSvc2-WT) and its fusion loop mutants for their abilities to release RSV virions from endosomes. In this system, purified RSV virions were incubated with a cell extract from Sf9 expressing NSvc2-WT for 3 h and then used it to feed SBPHs for 24 h. Midguts were isolated from the SBPHs and probed for the presence of RSV virions and NSvc2-N through immuno-labeling. Result showed that RSV virions (green) and NSvc2-N (red) were both present in the endosomal-like vesicles inside epithelial cells by 24 h post feeding (Fig 7A, upper row), indicating that both RSV virions and NSvc2-N had entered the midgut cells using this system. We then incubated purified RSV virions with cell extracts from Sf9 expressing the NSvc2^N114A/N199A/N232A^ or NSvc2^F460A/F489A/Y498A^ mutant and used them individually to feed SBPHs. The result showed that RSV virions were detected in the midgut lumen, but not in the epithelial cells, of the SBPHs fed with the mixture containing RSV virions and cell extract with the NSvc2^N114A/N199A/N232A^ mutant (Fig 7A, middle row). The result also showed that RSV virions were present in the epithelial cells and accumulated inside the endosomal-like structures of the SBPHs fed with the mixture containing RSV virions and cell extract with the NSvc2^F460A/F489A/Y498A^ mutant. In addition, more endosomal-like structures with RSV virions were found in the epithelial cells of the SBPHs fed with the mixture containing RSV virions and the NSvc2^F460A/F489A/Y498A^ mutant than that in the epithelial cells of the SBPHs fed with the mixture containing RSV virions and NSvc2-WT (Fig 7A, bottom row).

To investigate the effects of recombinant NSvc2-WT or its mutants on the acquisition and transmission of RSV by SBPH using the new developed system, the NSvc2-WT or its mutants were expressed from Sf9 cells, mixed with purified RSV virions, and then used to feed SBPHs. The results indicated that the SBPHs fed with the mixture containing RSV virions and NSvc2-WT had 12–18% RSV acquisition rate and 4–5% virus transmission rate (Fig 7B and S3 Table). For the SBPHs fed with the mixture containing RSV virions and the NSvc2^F460A/F489A/Y498A^ mutant, only 2–3% RSV acquisition rate and 0–1% virus transmission rate were observed. No RSV acquisition and transmission were found for the SBPHs fed with the mixture containing RSV virions and the NSvc2^N114A/N199A/N232A^ mutant. In the subsequent experiments, SBPHs were first fed with NSvc2-WT or its mutants and then RSV virions followed by the RSV acquisition and transmission assays. The results showed that the SBPHs fed with NSvc2-WT first and then RSV virions had 15–20% RSV acquisition rate and 6–8% virus transmission rate (Fig 7C and S4 Table). Only 4–5% RSV acquisition rate and 1–2% virus transmission rate were observed for the SBPHs fed with the NSvc2^F460A/F489A/Y498A^ mutant first and then RSV virions (Fig 7C and S4 Table). This result indicated that feeding SBPHs with NSvc2-WT first and then RSV virions could cause slightly higher virus acquisition and transmission rate compared with the SBPHs fed with the mixture containing both RSV virions and NSvc2-WT. Again, no RSV acquisition and transmission were observed for the SBPHs fed with the NSvc2^N114A/N199A/N232A^ mutant first and then RSV virions (Fig 7C and S4 Table). In a separate experiment, purified RSV virions were micro-injected into the hemocoel of SBPHs followed by RSV acquisition and transmission assays. The result showed that the micro-infected SBPHs had 66–72% RSV acquisition rate and 22–27% virus transmission rate (S5 Table), indicating that the midgut barrier(s) could be bypassed by micro-injecting RSV virions directly into SBPH hemocoel. Thus, the fusogenic activity deficient NSvc2 mutant is capable of passaging RSV virions through epithelial cells but is defective in releasing RSV virions from the endosomal-like vesicles.

## Discussion

Understanding how plant viruses are transmitted by their insect vectors is one of the key steps to manage virus diseases worldwide. Insect midgut is a major barrier to block the entrance of non-compatible plant viruses into insect vector. In this study, we used RSV and its SBPH vector as a working model to elucidate the molecular mechanism controlling RSV virion entrance into SBPH midgut cells for transmission. Through various assays, we have now determined that RSV, a circulative and propagative transmitted tenuivirus, utilizes its glycoprotein NSvc2 as a helper component to ensure the successful entrance of virions into the midgut of SBPH. This is the first evidence showing that tenuivirus uses a helper component mediated mechanism for persistent-propagative virus transmission.

Although the function of RSV glycoprotein NSvc2 during virus acquisition and transmission was previously proposed to be similar to the glycoproteins of other plant-infecting tospoviruses or animal-infecting bunyaviruses [31, 51, 52], our findings from this study showed that, unlike tospoviruses and bunyaviruses, RSV NSvc2 is not present on the surface of RSV non-enveloped filamentous virions (Fig 2B and Fig 3D–3F), and purified RSV virions were unable to enter SBPH midgut cells. The inability of RSV virions to enter the midgut cells could, however, be rescued by the addition of NSvc2 (Fig 2D and Fig 7). In addition, micro-injection of RSV virions directly into SBPH hemolymph could bypass the midgut barriers (S5 Table) [43]. More importantly, in the presence of NSvc2, purified RSV virions could be successfully acquired by SBPHs and then transmitted to rice seedlings (Fig 2C, Fig 7B and 7C). For non-persistent, semi-persistent, and persistent-nonpropagative transmitted plant viruses, the helper component proteins or the helper factors were shown to mediate the interactions between virus virions and their insect vectors [4, 6, 15, 17]. Although these helper component proteins or factors are not located on the surface of purified virions, they are absolutely required for insect transmissions [20, 53]. The results presented in this paper indicate that this previously proposed helper component theory can also be applied to explain the function of RSV NSvc2 during virus circulative and propagative transmission. First, the RSV NSvc2 is not a virion structural protein (Fig 2B and Fig 3). Second, NSvc2 can interact with RSV virions and bind to SBPH midgut (Fig 2E, and 2F and Fig 3). Third, NSvc2 is absolutely required for the entrance of RSV virions into SBPH midgut cells (Fig 2 and Fig 7). Moreover, the SBPHs fed first with the recombinant NSvc2 and then purified RSV virions had slightly higher RSV acquisition rate and virus transmission rate compared with the SBPHs fed with the recombinant NSvc2 and purified RSV virions simultaneously (Fig 7C). Consequently, we consider RSV NSvc2 as a helper component that is needed for the successful overcome of midgut barrier(s) in SBPH for virus persistent-propagative transmission. This helper component may function as a molecular bridge to ensure the proper interaction between RSV virions and SBPH midgut receptor(s). We, however, cannot rule out other alternative possibilities. Neither can we rule out the hypothesis that tenuiviruses can form membrane bound complexes with NSvc2, and these complexes are unstable during virus purification. However, earlier electron microscopic results using fixed tissue sections from RSV-infected rice leaves or RSV-infected SBPH tissues did not reveal the presence of membrane bound complexes in RSV-infected cells [54, 55].

RSV NSvc2 is known to be processed into NSvc2-N and NSvc2-C in the RSV-infected cells [41, 42]. We have found that NSvc2-N accumulated on the midgut surface during SBPH acquisition of RSV. The ectopically expressed soluble NSvc2-N:S could bind directly to the midgut surface and inhibit the subsequent RSV acquisition by SBPH. Our enzymatic deglycosylation results showed that NSvc2-N could be modified by N-linked but not O-linked glycans. The glycan-modification of NSvc2-N might be different from the N- or O-glycosylation of TSWV Gn protein reported previously [30]. It is noteworthy that the N-glycosylation deficient NSvc2-N^N114A/N199A/N232A^ mutant was unable to interact with midgut surface receptor(s) and was unable to block RSV acquisition and transmission by SBPH. In contrast to the recombinant NSvc2-WT purified from the Sf9 cells, the N-glycosylation deficient NSvc2 mutant was unable to mediate the entrance of RSV virions into SBPH midgut cells. The mutant failed to cause RSV acquisition and virus transmission by SBPH (Fig 7). Based on these results, we propose that N-glycosylation modification has an important role in the interaction between RSV NSvc2 and SBPH midgut surface. Sugar transporter 6 protein was recently reported to play a critical role in RSV invasion of SBPH midgut epithelial cells [56]. This sugar transporter interacted with RSV NP and was highly expressed in the midgut cells of SBPH. Expression of this protein in Sf9 cells allowed RSV virions to enter the cells [56]. In our study, RSV virions were detected only in the midgut lumen and not inside the epithelial cells of the SBPHs fed with purified RSV virions (Fig 2D, row 3). In the presence of NSvc2, however, RSV virions did enter the epithelial cells (Fig 2D and Fig 7), supporting the conclusion that NSvc2 is absolutely required for the passage of RSV virions through SBPH midgut barrier(s). We speculate that SBPH midgut may have more barriers for RSV virions entrance than that of the Sf9 cells. The outer capsids of rice black streak dwarf virus (RBSDV) and southern rice black streak dwarf virus (SRBSDV) were also shown to interact with SBPH sugar transporter 6 protein [56]. It would be interesting to see if NSvc2 can interact with SBPH sugar transporter 6 protein and if glycosylation of NScv2 plays an important role in the interaction between the two proteins.

Genomic RNAs of many animal-infecting viruses are encapsidated within bilayer lipid envelopes, which protect viruses during their transmissions between hosts. For bunyaviruses, the glycoprotein Gn and Gc on surface membrane envelopes are known to interact with viral ribonucleoprotein complexes (RNPs) which composed of the viral genomic RNAs and the nucleocapsid protein inside virions [51, 57]. Although the purified RSV virions are neither enveloped nor carrying the glycoprotein NSvc2 (Fig 3), the NSvc2 protein is associated with RSV virions during the entrance of virus into SBPH midgut. Using Yeast-Two-Hybrid and co-IP analysis, RSV glycoprotein NSvc2-N and NSvc2-C were also found to interact with RSV virions. These interactions are likely to form a stable RSV virion:NSvc2-N:NSvc2-C complexes during virus transmission by its SBPH vector. Endocytosis was shown to be important for the entrance of animal-infecting bunyaviruses into host cell [51, 58, 59]. Once inside a new host, the viral lipid envelopes fused with host cell membranes to release viral RNPs into the cytoplasm to start an infection [49, 58, 60]. After the recognition of RSV virions, midgut epithelial cells underwent endocytosis to compartmentalize RSV virions:NSvc2-N:NSvc2-C complexes in the early and then the late endosomes (Fig 5). After that, RSV virions were released from the late endosomes into the cytosol for further virus replication and spread into adjacent cells. Only RSV virions:NSvc2-N complexes were released into the cytosol, while the NSvc2-C was remained inside the endosomes. Because tenuivirus virions are not enveloped, it is unclear how membrane fusion affects the release of RSV virions into the cytosol. It was reported that acidic condition inside the late endosomes could trigger the conformation of bunyavirus Gc protein and induce membrane fusion that could disrupt the enveloped structure of virus virions and lead to the release of viral RNPs into the cytoplasm of host cell [50, 61]. It is possible that under the acidic condition, NSvc2-C also changes its structure and stops its association with RSV virions. Meanwhile, the membrane fusion triggered by NSvc2-C releases the RSV virions into the host cell cytosol.

Bunyavirus glycoprotein Gc was reported to be activated and to cause membrane fusion under the acidic conditions inside endosomes [62, 63]. We have also determined that under the acidic condition, NSvc2-C could cause Sf9 cell membrane to fuse (Fig 6). Crystal structures of several animal-infecting bunyavirus glycoprotein Gc have been determined and are considered as class II fusion proteins [61, 64, 65]. For the class II fusion proteins, the hydrophobic fusion loops were reported to be at the apex of domain II [64, 66]. For all known viral fusion proteins, the fusion loops are the key motifs that are inserted into the target membranes to bring the virus and target membranes together. Using a homology-based modeling approach, RSV NSvc2-C was also found to have a class-II fusion glycoprotein architecture with two putative fusion loops. Deletion of the two fusion loops resulted in a defect in membrane fusion. The two fusion loops found in NSvc2 are highly conserved among the members in the genus *Tenuivirus*, suggesting that they have conserved roles in membrane fusion. With the aid of homology-based modeling, we have determined that residue F460, F489 and Y498 in the fusion loop of NSvc2-C played critical roles in cell membrane fusion. Also, the NSvc2 mutant that failed to cause cell membrane fusion was unable to release RSV virions from endosomes into the cytosol. This mutant also caused much lower RSV acquisition rate and virus transmission rate by SBPH. Current gene function studies on plant multi-segmented negative-strand RNA viruses are difficult, due mainly to the lack of proper reverse genetics methods. In this study, we developed a new method to overcome this obstacle. We first expressed the WT or mutant NSvc2 in Sf9 insect cells, and then incubated the extracts from the infected Sf9 cells with purified RSV virions. After feeding SBPHs with a mixture containing purified RSV virions and NSvc2 or a mixture containing purified RSV virions and a mutant NSvc2, we have determined that in the presence of NSvc2, purified RSV virions were able to overcome the midgut barriers to enter earlier and late endosomes in the epithelial cells, and then be released from late endosomes to the cytosol for further virus replication and transmission. We have also been able to analyze the function of NSvc2 mutants during virus entrance to SBPH midgut cells. This new method should benefit gene function studies for viruses whose infectious clones are currently difficult to make. We think that this technology can not only be used to investigate the functions of tenuivirus glycoproteins but also the functions of glycoproteins encoded by other plant multi-segmented negative-strand RNA viruses.

**Fig 8:**
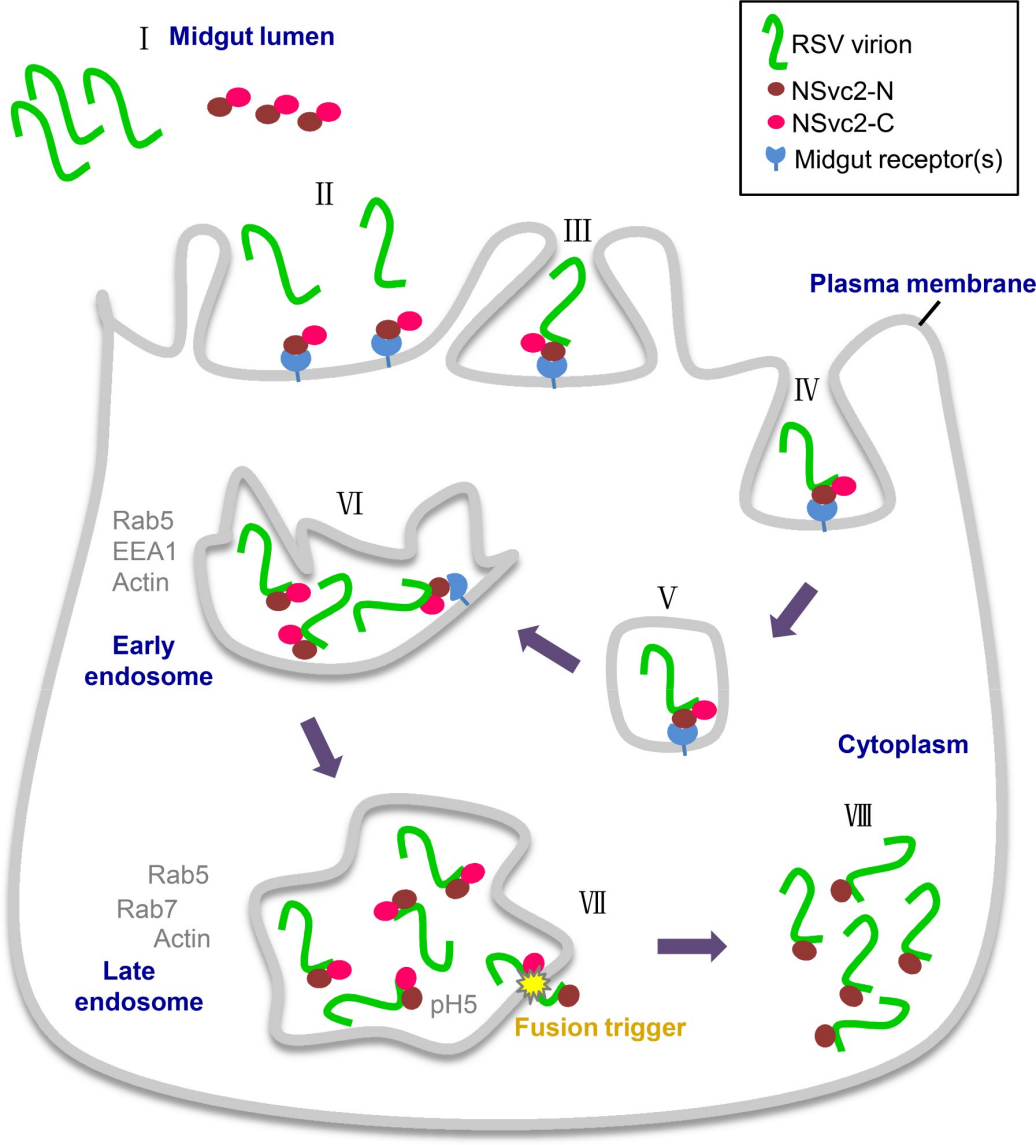
A helper component model for RSV virion entrance into SBPH midgut cells. During SBPH feeding on the RSV-infected rice plants, RSV virions, NSvc2-N and NSvc2-C are acquired into the midgut lumen (Step I). Inside the midgut lumen, NSvc2-N directs the virions:NSvc2-N:NSvc2-C complexes to microvillus surface through recognition of unidentified microvillus surface receptor(s) (Steps II and III). The complexes-attached midgut lumen membrane undergoes endocytosis and then compartmentalizes RSV virions:NSvc2-N:NSvc2-C complexes in vesicles (Steps IV and V). These vesicles further develop into early endosomes (Step VI) and then late endosomes (Step VII). The acidic condition inside the late endosomes causes the conformation change of NSvc2-C, leading to cell-cell membrane fusion (Step VII). Finally, the RSV virions:NSvc2-N complexes are released from the late endosomes into the cytosol of epithelial cells for further RSV replication and spread between midgut cells (Step VIII).

Taken together, we conclude that the circulative and propagative transmitted RSV uses a helper component strategy to ensure the entrance of RSV virions into SBPH midgut during vector transmission. Based on the findings presented in this paper, we propose a working model for deciphering how plant-infecting tenuiviruses overcome the SBPH midgut barriers (Fig 8). In this model, plant sap containing RSV virions, NSvc2-N, and NSvc2-C is acquired into the midgut lumen during SBPH feeding on the RSV-infected rice plants. The NSvc2-N protein recognizes the unidentified midgut cell surface receptor(s) and acts as a helper component to ensure the interaction between RSV virions and midgut surface receptor(s). Upon attachment of RSV virions:NSvc2-N:NSvc2-C complexes to the midgut surface receptor(s), midgut cells undergo endocytosis, resulting in compartmentalization of RSV virions:NSvc2-N:NSvc2-C complexes in the early and then in the late endosomes. The acidic condition inside the late endosomes triggers a conformation change of NSvc2-C, and the conformation-changed NSvc2-C causes the membrane fusion. Finally, the RSV virions:NSvc2-N complexes are released from endosomes into the cytosol. The findings presented in this paper revealed a new type of virus–insect midgut interaction that requires a virally encoded helper component during virus persistent-propagative transmission.

## Materials and methods

### Virus and insect maintenance

Rice stripe virus was previously isolated from an RSV-infected rice plant and then maintained inside a growth chamber at the Jiangsu Academy of Agricultural Sciences, Nanjing, Jiangsu Province, China. Small brown planthopper (SBPH) was reared on rice seedlings cv. Wuyujing NO.3 inside growth incubators set at 26.5°C and a photoperiod of 16 h / 8 h (light / dark). Rice seedlings were changed once every 12 days as described [67].

### Purification of RSV virions

Eight hundred milliliter precooled 0.1 M phosphate buffer (PB), pH 7.5, with 0.01 M EDTA was added to 50-gram RSV-infected rice leaf tissues followed by 5 min homogenization in a blender. The homogenate was centrifuged at 8,000 × *g* for 20 min at 4°C. The resulting supernatant was mixed with PEG 6000 (6%) and NaCL (0.1 M), and then stirred overnight at 4°C. After centrifugation at 8,000 × *g* for 20 min, the pellet was resuspended in 0.01 M PB followed by 2 h centrifugation at 150,000 × *g*. The pellet was resuspended in 6 ml 0.01 M PB and laid on the top of a 4 ml 20% glycerol cushion inside a centrifuge tube followed by a centrifugation at 150,000 × *g* for 2 h. Different fractions inside the centrifuge tube were carefully collected and the pellet was resuspended in 4 mL PB buffer containing 30% glycerol. The collected samples were stored at -70°C till use.

### Immunolabeling of SBPH midguts

Immunofluorescence labeling was performed as described previously with specific modifications [45]. Midguts were obtained from second-instar nymphs and fixed overnight in a 4% paraformaldehyde (PFA) (Thermo Fisher Scientific, Waltham, MA USA) solution at 4°C. After three rinses in a 0.01 M phosphate-buffered saline (PBS), pH 7.4, the midguts were treated for 30 min in a 2% Triton X-100 solution followed by 1 h incubation in a 1:200 (v/v) diluted specific primary antibody. The midguts were then incubated in a 1:200 (v/v) diluted specific fluorescence conjugated secondary antibody for 2 h at room temperature (RT). The midguts were rinsed three times in the PBS and then mounted in an antifade solution (Solarbio, Shanghai, China). The mounted midguts were examined under an inverted Leica TCS SP8 fluorescent confocal microscope (Leica Microsystems, Solms, Germany). Images of single sections were taken to show the co-localizations of two assayed proteins. The overlapped coefficient value was determined using the LAS X software, and 50 merged spots from 30 guts per experiment were analyzed.

Rabbit polyclonal antibodies against RSV NSvc2-N or NSvc2-C were produced in our laboratory. Mouse monoclonal antibody against RSV NP was a gift from Professor Jianxiang Wu, Zhejiang University, Hangzhou, China. Rab5 anti-rabbit IgG and Rab7 anti-rabbit IgG were from the Cell Signaling Technology (Danvers, MA, USA), and EEA1 anti-mouse IgG was from Novus (Centennial, CO, USA). Secondary antibodies used in this study were FITC conjugated rabbit anti-mouse IgG (F9137) or goat anti-rabbit IgG (F9887), and TRITC conjugated goat anti-rabbit IgG (T6778) from Sigma-Aldrich (St. Louis, MO, USA). Alexa Fluor 647 phalloidin (A22287) and Rhodamine Phalloidin (R415) were from Invitrogen (Carlsbad, CA, USA). These antibodies were used at 1:200 (v/v) dilution.

### Immunogold labeling of RSV virions

Formvar/carbon-coated nickel grids (200 mesh) were floated individually on drops of PB buffer containing purified RSV virions for 2 min. The grids were then transferred onto drops of 1% BSA buffer and incubated for 10 min followed by 1 h incubation on drops of 1:200 (v/v) diluted mouse monoclonal antibody against RSV NP or rabbit polyclonal antibody against NSvc2-N. The grids were rinsed with several drops of PB and then incubated on drops of 1:30 (v/v) diluted goat anti-mouse IgG conjugated with 15 nm gold particles or goat anti-rabbit IgG conjugated with 8 nm gold particles for 1 h. After several rinses with PB, the grids were stained with 2% uranyl acetate and then examined under a Hitachi HT-7700 transmission electron microscope.

### Expression and purification of NSvc2 proteins in Sf9 insect cells

Preparation of recombinant baculoviruses was as described previously [68]. Sequence encoding RSV NSvc2, NSvc2-N, NSvc2-C, NSvc2-N:S (amino acid position 20 to 265, lacking the signal peptide and the transmembrane domain), and TSWV Gn:S was PCR-amplified using a cDNA made from an RSV-infected rice plant or from a TSWV-infected *Nicotiana benthamiana* plant using specific primers (S6 Table). The NSvc2-N and NSvc2-C contained a FLAG tag and the NSvc2-N:S contained a six-His tag sequence at their 3' end. A Gp64 signal peptide sequence was then fused to the 5' end of RSV NSvc2 or TSWV Gn:S proteins via overlapping PCR and the product was cloned into vector pFastBac1 (S6 Table). The site-directed mutagenized mutants were constructed as described [69]. All plasmids were sequenced and then transformed individually into DH10Bac^TM^ cells to generate recombinant baculoviruses as instructed by the manufacturer (Invitrogen). The recombinant baculoviruses were mixed individually with the FnGENE HD Transfection Reagent (Promega) and infected Sf9 cells as instructed.

Sf9 cells were infected with various recombinant baculoviruses at the multiplicity of infection (MOI) of five. The infected Sf9 cells were collected after 72 h incubation and were lysed in 10 ml PBS buffer using an ultrasonic cell crusher followed by centrifugation at 4°C to remove cell debris. Supernatant from individual treatment was collected, incubated with a nickel-nitrilotriacetic acid resin (Ni-NTA, Germany) for 2 h, and then loaded onto a chromatographic column (Bio-Rad Hercules, California, USA). After separation, the column was washed with two bed volumes of 50 mM imidazole in PBS, and the recombinant proteins were then eluted from the columns with 250 mM imidazole solution followed by dialysis against PBS. The purified recombinant proteins were stored at -70°C until use.

### Glycosylation and immunoblotting assays

Purified NSvc2-N:S and other mutant proteins were individually deglycosylated with PNGaseF to remove the N-linked glycans or with Neuraminidase and O-Glycosidase to remove the O-linked glycans as instructed (New England Biolabs, Ipswich, MA, USA). Sodium dodecyl sulfate (SDS) and dithiothreitol (DTT) were added to the purified protein samples, respectively, and the mixed samples were heated at 100°C for 10 min. After denaturation, buffer and glycosidases were added to the samples followed by 3 h incubation at 37°C. The enzyme-treated samples were mixed with a loading buffer containing SDS and boiled for 10 min prior to electrophoresis in 10% (w/v) SDS-PAGE gels. The separated proteins were transferred onto nitrocellulose membranes and the membranes were probed individually with the NSvc2-N antibody diluted at 1:5,000 (v/v) followed by a goat anti-rabbit IgG HRP conjugate (31466, Invitrogen) diluted at 1:10,000 (v/v). The detection signal was visualized using the ChemiDoc Touch Imaging System as instructed (Bio-Rad).

### Glycoprotein midgut binding assays

Midgut binding assays were performed as described previously [30]. Briefly, second-instar nymphs were placed inside open-ended Eppendorf centrifuge tubes and fed with purified glycoproteins resuspended in a TF buffer (PBS with 10% glycerol, 0.01% Chicago sky blue, and 5 mg / ml BSA) through a stretched parafilm membrane. After 3 h feeding, the glycoprotein samples were replaced with a 10% sucrose solution and the insects were allowed to feed for another 12 h to clear their midguts, indicated by the disappearance of the Chicago sky blue dye from the midguts. The insects were then dissected, fixed in a 4% paraformaldehyde solution and analyzed for the binding through immunofluorescence labeling as described above.

### Detection of RSV acquisition and transmission by SBPH using immuno-dot blot assays

Second-instar nymphs were placed inside empty bottles for 2 h and then allowed to feed on purified recombinant NSvc2-N:S protein or mutant proteins through stretched parafilm membranes for 24 h. The pre-fed SBPHs were then allowed to feed on RSV-infected rice seedlings for 48 h. Second-instar nymphs were also fed on a purified RSV virion, supernatanta mixture of both or mixture of purified RSV virions and Sf9 cell extract containing NSvc2 proteins through stretched parafilm membranes for 24 h followed by 12 day feeding on healthy rice seedlings. After that, the SBPHs were individually transferred onto individual healthy rice seedlings cv. Wuyujing NO.3 inside glass tubes for virus transmission test for 24 h. After virus transmission, the seedlings were allowed to grow for 14 days prior to RSV infection test. One hundred SBPHs and one hundred rice seedlings were used for each treatment. In a separate experiment, purified RSV virions (about 70 ng/μl) were micro-injected into the hemocoel of SBPHs using a microprocessor-controlled Nanoliter 2010 injector (World Precision Instruments, Sarasota, FL, USA). The RSV acquisition and virus transmission of the injected SBPHs were performed using the same methods as above.

The rates of RSV acquisition and transmission by SBPHs were determined by immuno-dot blot assays. SBPHs were collected individually at 12 days post 48 h feeding on the RSV-infected rice seedlings or on a purified RSV virion sample, or at 14 days post 24 h feeding on the purified RSV viroins, recombinant protein or a mixture of both. The collected SBPHs were grinded individually in PBS buffer. The samples were centrifuged at 5,000 rpm for 1 min at 4°C to remove cell debris and the supernatant from each sample was blotted onto nitrocellulose membranes. The membranes were probed with a 1:5,000 (v/v) diluted RSV NP antibody and then with a 1:20,000 diluted secondary alkaline phosphatase (AP)-conjugated goat anti-mouse IgG (Sigma). The detection signal was visualized using a 5-bromo-4-chloro-3-indo-lylphosphate-nitroblue tetrazolium (BCIP-NBT) solution (Sangon Biotech, Shanghai, China). Three independent experiments with 50 nymphs per treatment were performed.

### Quantitative real-time PCR

Total RNA was isolated from individual SBPHs using TRIzol (Thermo Fisher Scientific). cDNAs were synthesized using the M-MLV reverse transcriptase (M1701, Promega). Quantitative PCR was performed on a CFX Connect Real-Time System (Bio-Rad, USA) using the PowerUp SYBR Green Master Mix (A25742, Thermo Fisher Scientific). RSV specific primers were designed using the Primer Premier 5.0 software. The expression level of SBPH *Actin* gene was used as an internal reference. The relative abundance of RSV RNA in SBPHs was calculated using the 2 –^ΔΔCt^ method. Primers used for the RT-qPCR are listed in S6 Table.

### Yeast two-hybrid assay

The coding sequence of RSV NSvc2-N or NSvc2-C was cloned into a bait vector pGBKT7. The coding sequence of RSV NP was cloned into a prey vector pGADT7. Primers used in PCR reactions are listed in S6 Table. Yeast two-hybrid assays were performed as previously described [70, 71]. Briefly, a bait vector and a prey vector (see details in the result section) were co-transformed into the Y2HGold strain cells by heat shock method. In addition, vector pGADT7-T was co-transformed with vector pGBKT7-53 or vector pGBKT7-Lam into the Y2HGold strain cells and the cells were used as the positive and the negative control, respectively. All the cells were first grown on a synthetic dextrose medium lacking Tryptophan and Leucine amino acid (SD-Trp-Leu) for 3 days at 30°C, and then on a synthetic dextrose medium lacking Tryptophan, Leucine, Histidine and Ademethionine amino acid (SD-Trp-Leu-His-Ade) for 5 days at 30°C.

### Low pH-induced cell membrane fusion assay

Sf9 cells were transfected with different recombinant baculoviruses at a MOI of five. After 48 h infection, the infect cells were washed twice with a fresh medium, treated in a PBS buffer, pH 5.0, for 2 min, and then grown in a pH neutral medium for 4 h at 28°C. The cell was then examined for cell-cell membrane fusion under light/bright bield ofan Olympus IX71 inverted microscope (Olympus, Hamburg, Germany).

### Homology modeling of NSvc2-C

Homology modeling of NSvc2-C was as described previously [72, 73]. Briefly, RSV NSvc2-C sequence was used to search the I-TASSER Server (https://zhanglab.ccmb.med.umich.edu/I-TASSER/). Based on the high TM-score value (0.805), glycoprotein of *Sever Fever with Thrombocytopenia Syndrome Phlebovirus* (SFTSV) (PDB: 5G47) was chosen as the template to build the homology-based model of RSV NSvc2-C. Amino acid residues and their surface positions in the three-dimensional structure were predicted using the PyMOL program (https://pymol.org/2/).

## Supporting information

S1 Fig Quantification of co-localization of RSV virions and NSvc2 in SBPH midgut. (A) Quantification of co-localization of RSV virions and NSvc2 in SBPH midgut at 4, 8, 16 and 24 h post feeding on RSV-infected rice plants. (B) Quantification of co-localization of RSV virions and NSvc2 in SBPH midgut after feeding on different centrifugation fractions. Fifty fluorescent spots were randomly selected from 30 guts per experiment and analyzed for co-localization of RSV virions and NSvc2. Sup, supernatant fractions; Gly, glycerol fractions; Pel, resuspended pellet sample.
(PDF)

S2 Fig Negative controls for detections of NSvc2 and RSV virions in SBPH midgut cells. (A and B) Immunofluorescence labeling of NSvc2 and RSV virions on the surface of SBPH intestinal microvillus (blue) at 8 h (A) and 24 h (B) post feeding on the healthy rice plants. (C) Immunofluorescence labeling of NSvc2 and RSV virions on the surface of SBPH intestinal microvillus at 24 h post feeding on the mixed glycerol fractions prepared from the healthy rice plants. The samples were probed with the NSvc2-N (red), RSV NP (green), or actin (blue) specific antibody. Bar, 25 μm. The overlap fluorescence spectra from NSvc2 and RSV virion labelings at different stages were determined using the white dashed line and shown right.
(PDF)

S3 Fig Organizations of the full length NSvc2 and its recombinant soluble N-terminal region (NSvc2-N:S). (A) A diagram of NSvc2 with different domains and putative glycosylation sites. SP, signal peptide; TM, transmembrane domain. (B) A diagram of NSvc2-N:S with different domains and putative glycosylation sites. The signal peptide of NSvc2-N:S is replaced with a Gp64 signal peptide. (C) Detection of NSvc2-N:S expression in Sf9 cells using a NSvc2-N specific antibody. Protein marker sizes are indicated on the left side and the labeled NSvc2-N:S band is indicated with an arrow.
(PDF)

S4 Fig Pre-binding of recombinant soluble NSvc2-N to midgut inhibited subsequent passages of RSV virions into midgut epithelial cells. (A-C) Effects of pre-feeding with purified NSvc2-N:S (A), TSWV Gn:S (B) and sucrose alone (C) on RSV virion entrance into SBPH midguts. The boxed regions are enlarged and shown on the right side. The overlap fluorescence spectra were from the white dashed line indicated areas. (D) Percentages of RSV virion invaded SBPH midgut epithelial cells. **, *p* < 0.01 by the student *t*-test. EC, epithelial cells; Bar, 25 μm.
(PDF)

S5 Fig Time course study of NSvc2-C accumulation during RSV entrance into SBPH midgut cells. (A) NSvc2-C and RSV virions were co-localized on the surface of midgut microvillus (blue) at 4 h post feeding on the RSV-infected rice seedlings. The boxed region was enlarged and shown with three panels on the right side. The labeled NSvc2-C is shown in red and the labeled RSV virions are shown in green. (B) NSvc2-C and RSV virions were co-localized in the endosomal-like vesicles inside the midgut epithelial cells at 8 h post feeding. (C) RSV virions were detected in the cytosol of epithelial cells at 16 h post feeding but not NSvc2-C. (D) NSvc2-C was not detected in the cytosol with RSV virions at 24 h post feeding. (E–H) Analyses of overlap fluorescence spectra from the white dashed line indicated regions in the merged images. The overlap coefficient (OC) values were determined using the LAS X software. ML, midgut lumen; EC, epithelial cells; Bar, 10 μm.
(PDF)

S6 Fig Hydrophobic fusion-loop motifs in NSvc2-C are required for cell-cell membrane fusion. (A) A diagram showing different domains in NSvc2-C. Domain I is in yellow, domain II is in red and domain III is in blue. Fusion loops (FL1 and FL2) are shown in green and a hydrophobic region in gray. (B) A three-dimensional homology structure model of NSvc2-C, with the same color arrangement as shown in (A). (C) Expressions of the FLAG-tagged NSvc2-N or NSvc2-C in the Sf9 cells were confirmed by Western blot assays. The NSvc2-N-FLAG and NSvc2-C-FLAG proteins were enriched individually and then detected using an anti-FLAG antibody. Arrows indicate the bands of the expressed NSvc2-N-FLAG (40 kDa) or NSvc2-C-FLAG (50 kDa) proteins. (D) Schematic representations of NSvc2-C and its FL deletion mutants. Locations of FL1 (black) and FL2 (gray) are shown. Deletions of one fusion loop (ΔFL1 or ΔFL2) or both fusion loops (ΔFL1+ΔFL2) are indicated with upward open arrows. (E) Expressions of the FLAG-tagged NSvc2-C and its FL deletion mutants in the Sf9 cells were determined by immunoblotting. The blots were probed with a FLAG tag-specific antibody. Arrow indicates the bands of the expressed proteins. The empty vector (Vec) was used as a negative control. (F and G) Analyses of fusogenic activities of NSvc2-C and its FL deletion mutants. Sf9 cells were infected with the recombinant baculoviruses expressing NSvc2-C or one of its deletion mutants. At 48 h post infection, the Sf9 cells were treated for membrane fusion assays (F). The numbers of cells per syncytium were counted and analyzed (G). The experiment was repeated three times. **, *p* < 0.01 by the student *t*-test.
(PDF)

S1 Table RSV acquisition and transmission efficiency by SBPHs fed on the combined supernatant fractions (Sup), combined glycerol fractions (Gly), the resuspended pellet (Pel) sample or a combination of Sup and Pel. (DOCX)

S2 Table RSV acquisition and transmission efficiency by SBPHs pre-fed with NSvc2-N:S or its mutant proteins followed by feedings on the RSV-infected rice seedlings. (DOCX)

S3 Table RSV acquisition and transmission efficiency by SBPHs fed with a mixture of purified RSV virions and NSvc2, or a mixture of purified RSV virions and an NSvc2 mutant. (DOCX)

S4 Table RSV acquisition and transmission efficiency by SBPHs pre-fed with NSvc2 or one of its mutants followed by feedings on purified RSV virions. (DOCX)

S5 Table RSV acquisition and transmission efficiency by SBPHs micro-injected with the combined glycerol fractions, the resuspended pellet sample or sucrose solution. (DOCX)

S6 Table Primers used in this study. (XLSX)

## References

[1] Gray SM, Banerjee N. Mechanisms of arthropod transmission of plant and animal viruses. Microbiol Mol Biol R. 1999; 63:128–48. . 10066833 10.1128/mmbr.63.1.128-148.1999 PMC98959

[2] Ruckert C, Weger-Lucarelli J, Garcia-Luna SM, Young MC, Byas AD, Murrieta RA, et al Impact of simultaneous exposure to arboviruses on infection and transmission by Aedes aegypti mosquitoes. Nat commun. 2017; 8: 15412 10.1038/ncomms15412 . 28524874 PMC5454532

[3] Dader B, Then C, Berthelot E, Ducousso M, Ng JCK, Drucker M. Insect transmission of plant viruses: Multilayered interactions optimize viral propagation. Insect Sci. 2017; 24: 929–46. 10.1111/1744-7917.12470 . 28426155

[4] Ng JC, Falk BW. Virus-vector interactions mediating nonpersistent and semipersistent transmission of plant viruses. Annu Rev Phytopathol. 2006; 44: 183–212. 10.1146/annurev.phyto.44.070505.143325 . 16602948

[5] Hogenhout SA, Ammar ED, Whitfield AE, Redinbaugh MG. Insect vector interactions with persistently transmitted viruses. Annu Rev Phytopathol. 2008; 46: 327–59. 10.1146/annurev.phyto.022508.092135 . 18680428

[6] Whitfield AE, Falk BW, Rotenberg D. Insect vector-mediated transmission of plant viruses. Virology. 2015; 479: 278–89. 10.1016/j.virol.2015.03.026 . 25824478

[7] Childress SA, Harris KF. Localization of Virus-Like Particles in the Foreguts of Viruliferous Graminella-Nigrifrons Leafhoppers Carrying the Semi-Persistent Maize Chlorotic Dwarf Virus. J Gen Virol. 1989; 70: 247–51. 10.1099/0022-1317-70-1-247

[8] Martin B, Collar JL, Tjallingii WF, Fereres A. Intracellular ingestion and salivation by aphids may cause the acquisition and inoculation of non-persistently transmitted plant viruses. J Gen Virol. 1997; 78: 2701–5. 10.1099/0022-1317-78-10-2701 . 9349493

[9] Chen AY, Walker GP, Carter D, Ng JC. A virus capsid component mediates virion retention and transmission by its insect vector. Proc Natl Acad Sci USA. 2011; 108: 16777–82. 10.1073/pnas.1109384108 . 21930903 PMC3189079

[10] Maluta NKP, Garzo E, Moreno A, Navas-Castillo J, Fiallo-Olive E, Lopes JRS, et al Stylet penetration activities of the whitefly Bemisia tabaci associated with inoculation of the crinivirus Tomato chlorosis virus. J Gen Virol. 2017; 98: 1515–20. 10.1099/jgv.0.000783 . 28613151

[11] Blanc S, Drucker M, Uzest M. Localizing viruses in their insect vectors. Annu Rev Phytopathol. 2014; 52: 403–25. 10.1146/annurev-phyto-102313-045920 . 24996011

[12] Gray S, Cilia M, Ghanim M. Circulative, "nonpropagative" virus transmission: an orchestra of virus-, insect-, and plant-derived instruments. Adv Virus Res. 2014; 89: 141–99. 10.1016/B978-0-12-800172-1.00004-5 . 24751196

[13] Pirone TP, Blanc S. Helper-dependent vector transmission of plant viruses. Annu Rev Phytopathol. 1996; 34: 227–47. 10.1146/annurev.phyto.34.1.227 . 15012542

[14] Ng JCK, Zhou JS. Insect vector-plant virus interactions associated with non-circulative, semi-persistent transmission: current perspectives and future challenges. Curr Opin Virol. 2015; 15: 48–55. 10.1016/j.coviro.2015.07.006 . 26318639

[15] Franz AWE, van der Wilk F, Verbeek M, Dullemans AM, van den Heuvel JFJM. Faba bean necrotic yellows virus (genus Nanovirus) requires a helper factor for its aphid transmission. Virology. 1999; 262: 210–9. 10.1006/viro.1999.9904 . 10489354

[16] Uzest M, Gargani D, Drucker M, Hebrard E, Garzo E, Candresse T, et al A protein key to plant virus transmission at the tip of the insect vector stylet. Proc Natl Acad Sci USA. 2007; 104: 17959–64. 10.1073/pnas.0706608104 . 17962414 PMC2084279

[17] Webster CG, Pichon E, van Munster M, Monsion B, Deshoux M, Gargani D, et al Identification of Plant Virus Receptor Candidates in the Stylets of Their Aphid Vectors. J Virol. 2018; 92: e00432–18. 10.1128/JVI.00432-18 . 29769332 PMC6026765

[18] Atreya CD, Pirone TP. Mutational Analysis of the Helper Component-Proteinase Gene of a Potyvirus—Effects of Amino-Acid Substitutions, Deletions, and Gene Replacement on Virulence and Aphid Transmissibility. Proc Natl Acad Sci USA. 1993; 90: 11919–23. 10.1073/pnas.90.24.11919 . 8265648 PMC48096

[19] Blanc S, Ammar ED, Garcia-Lampasona S, Dolja VV, Llave C, Baker J, et al Mutations in the potyvirus helper component protein: effects on interactions with virion and aphid stylets. J Gen Virol. 1998; 79: 3119–22. 10.1099/0022-1317-79-12-3119 . 9880030

[20] Ruiz-Ferrer V, Boskovic J, Alfonso C, Rivas G, Llorca O, Lopez-Abella D, et al Structural analysis of tobacco etch potyvirus HC-Pro oligomers involved in aphid transmission. J Virol. 2005; 79: 3758–65. 10.1128/JVI.79.6.3758-3765.2005 . 15731269 PMC1075709

[21] Lung MC, Pirone TP. Acquisition factor required for aphid transmission of purified cauliflower mosaic virus. Virology. 1974; 60: 260–4. . 4841178 10.1016/0042-6822(74)90383-3

[22] Leh V, Jacquot E, Geldreich A, Hermann T, Leclerc D, Cerutti M, et al Aphid transmission of cauliflower mosaic virus requires the viral PIII protein. EMBO J. 1999; 18: 7077–85. 10.1093/emboj/18.24.7077 . 10601029 PMC1171770

[23] Drucker M, Froissart R, Hebrard E, Uzest M, Ravallec M, Esperandieu P, et al Intracellular distribution of viral gene products regulates a complex mechanism of cauliflower mosaic virus acquisition by its aphid vector. Proc Natl Acad Sci USA. 2002; 99: 2422–7. 10.1073/pnas.042587799 . 11842201 PMC122380

[24] Brault V, Vandenheuvel JFJM, Verbeek M, Zieglergraff V, Reutenauer A, Herrbach E, et al Aphid Transmission of Beet Western Yellows Luteovirus Requires the Minor Capsid Read-through Protein P74. EMBO J. 1995; 14: 650–9. . 7882968 10.1002/j.1460-2075.1995.tb07043.x PMC398128

[25] Gray S, Gildow FE. Luteovirus-aphid interactions. Annu Rev Phytopathol. 2003; 41: 539–66. 10.1146/annurev.phyto.41.012203.105815 . 12730400

[26] Azzam O, Frazer J, Delarosa D, Beaver JS, Ahlquist P, Maxwell DP. Whitefly transmission and efficient ssDNA accumulation of bean golden mosaic geminivirus require functional coat protein. Virology. 1994; 204: 289–96. 10.1006/viro.1994.1533 . 8091659

[27] Noris E, Vaira AM, Caciagli P, Masenga V, Gronenborn B, Accotto GP. Amino acids in the capsid protein of tomato yellow leaf curl virus that are crucial for systemic infection, particle formation, and insect transmission. J Virol. 1998; 72: 10050–7. . 9811744 10.1128/jvi.72.12.10050-10057.1998 PMC110531

[28] Tomaru M, Maruyama W, Kikuchi A, Yan J, Zhu Y, Suzuki N, et al The loss of outer capsid protein P2 results in nontransmissibility by the insect vector of rice dwarf phytoreovirus. J Virol. 1997; 71: 8019–23. . 9311898 10.1128/jvi.71.10.8019-8023.1997 PMC192165

[29] Wei TY, Li Y. Rice Reoviruses in Insect Vectors. Annu Rev Phytopathol. 2016; 54: 99–120. 10.1146/annurev-phyto-080615-095900 . 27296147

[30] Whitfield AE, Ullman DE, German TL. Expression and characterization of a soluble form of Tomato spotted wilt virus glycoprotein G(N). J Virol. 2004; 78: 13197–206. 10.1128/JVI.78.23.13197-13206.2004 . 15542672 PMC524983

[31] Sin SH, McNulty BC, Kennedy GG, Moyer JW. Viral genetic determinants for thrips transmission of Tomato spotted wilt virus. Proc Natl Acad Sci USA. 2005; 102: 5168–73. 10.1073/pnas.0407354102 . 15753307 PMC552972

[32] Redinbaugh MG, Hogenhout SA. Plant rhabdoviruses. Curr Top Microbiol Immunol. 2005; 292: 143–63. . 15981471 10.1007/3-540-27485-5_7

[33] Ammar ED, Tsai CW, Whitfield AE, Redinbaugh MG, Hogenhout SA. Cellular and Molecular Aspects of Rhabdovirus Interactions with Insect and Plant Hosts. Annu Rev Entomol. 2009; 54: 447–68. 10.1146/annurev.ento.54.110807.090454 . 18793103

[34] Hibino H. Biology and epidemiology of rice viruses. Annu Rev Phytopathol. 1996; 34: 249–74. 10.1146/annurev.phyto.34.1.249 . 15012543

[35] Cho WK, Lian S, Kim SM, Park SH, Kim KH. Current Insights into Research on Rice stripe virus. Plant Pathol J. 2013; 29: 223–33. 10.5423/PPJ.RW.10.2012.0158 . 25288949 PMC4174810

[36] Cifuentes-Munoz N, Salazar-Quiroz N, Tischler ND. Hantavirus Gn and Gc Envelope Glycoproteins: Key Structural Units for Virus Cell Entry and Virus Assembly. Viruses. 2014; 6: 1801–22. 10.3390/v6041801 . 24755564 PMC4014721

[37] Spiegel M, Plegge T, Pohlmann S. The Role of Phlebovirus Glycoproteins in Viral Entry, Assembly and Release. Viruses. 2016; 8: 202–21. 10.3390/v8070202 . 27455305 PMC4974537

[38] Ramirez BC, Haenni AL. Molecular biology of tenuiviruses, a remarkable group of plant viruses. J Gen Virol. 1994; 75: 467–75. 10.1099/0022-1317-75-3-467 . 8126445

[39] Falk BW, Tsai JH. Biology and molecular biology of viruses in the genus Tenuivirus. Annu Rev Phytopathol. 1998; 36: 139–63. 10.1146/annurev.phyto.36.1.139 . 15012496

[40] Huo Y, Chen LY, Su L, Wu Y, Chen XY, Fang RX, et al Artificial feeding Rice stripe virus enables efficient virus infection of Laodelphax striatellus. J Virol Methods. 2016; 235: 139–43. 10.1016/j.jviromet.2016.06.003 . 27283882

[41] Zhao S, Zhang G, Dai X, Hou Y, Li M, Liang J, et al Processing and intracellular localization of rice stripe virus Pc2 protein in insect cells. Virology. 2012; 429: 148–54. 10.1016/j.virol.2012.04.018 . 22575054

[42] Yao M, Liu XF, Li S, Xu Y, Zhou YJ, Zhou XP, et al Rice stripe tenuivirus NSvc2 glycoproteins targeted to the golgi body by the N-terminal transmembrane domain and adjacent cytosolic 24 amino acids via the COP I- and COP II-dependent secretion pathway. J Virol. 2014; 88: 3223–34. 10.1128/JVI.03006-13 . 24390331 PMC3957912

[43] Toriyama S. Characterization of Rice Stripe Virus: a heavy component carrying infectivity. J Gen Virol. 1982; 61: 187–95. 10.1099/0022-1317-61-2-187

[44] Toriyama S. An RNA-dependent RNA-polymerase associated with the filamentous nucleoproteins of rice stripe virus. J Gen Virol. 1986; 67: 1247–55. 10.1099/0022-1317-67-7-1247

[45] Chen Q, Chen HY, Mao QZ, Liu QF, Shimizu T, Uehara-Ichiki T, et al Tubular Structure Induced by a Plant Virus Facilitates Viral Spread in Its Vector Insect. PLoS pathogens. 2012; 8:e1003032 10.1371/journal.ppat.1003032 . 23166500 PMC3499585

[46] Mercer J, Schelhaas M, Helenius A. Virus entry by endocytosis. Annu Rev Biochem. 2010; 79: 803–33. 10.1146/annurev-biochem-060208-104626 . 20196649

[47] Saeed MF, Kolokoltsov AA, Albrecht T, Davey RA. Cellular entry of ebola virus involves uptake by a macropinocytosis-like mechanism and subsequent trafficking through early and late endosomes. PLoS Pathog. 2010; 6: e1001110 10.1371/journal.ppat.1001110 . 20862315 PMC2940741

[48] Xia WQ, Liang Y, Chi Y, Pan LL, Zhao J, Liu SS, et al Intracellular trafficking of begomoviruses in the midgut cells of their insect vector. PLoS Pathog. 2018; 14: e1006866 10.1371/journal.ppat.1006866 . 29370296 PMC5800681

[49] Podbilewicz B. Virus and cell fusion mechanisms. Annu Rev Cell Dev Biol. 2014; 30: 111–39. 10.1146/annurev-cellbio-101512-122422 . 25000995

[50] Halldorsson S, Behrens AJ, Harlos K, Huiskonen JT, Elliott RM, Crispin M, et al Structure of a phleboviral envelope glycoprotein reveals a consolidated model of membrane fusion. Proc Natl Acad Sci USA. 2016; 113: 7154–9. 10.1073/pnas.1603827113 . 27325770 PMC4932967

[51] Lozach PY, Mancini R, Bitto D, Meier R, Oestereich L, Overby AK, et al Entry of Bunyaviruses into Mammalian Cells. Cell Host Microbe. 2010; 7: 488–99. 10.1016/j.chom.2010.05.007 . 20542252 PMC7172475

[52] Ruckert C, Ebel GD. How Do Virus-Mosquito Interactions Lead to Viral Emergence? Trends Parasitol. 2018; 34: 310–21. 10.1016/j.pt.2017.12.004 . 29305089 PMC5879000

[53] Hoh F, Uzest M, Drucker M, Plisson-Chastang C, Bron P, Blanc S, et al Structural insights into the molecular mechanisms of cauliflower mosaic virus transmission by its insect vector. J Virol. 2010; 84: 4706–13. 10.1128/JVI.02662-09 . 20181714 PMC2863735

[54] Liang D, Qu Z, Ma X, Hull R. Detection and localization of Rice stripe virus gene products in vivo. Virus genes. 2005; 31: 211–21. 10.1007/s11262-005-1798-6 . 16025247

[55] Deng J, Li S, Hong J, Ji Y, Zhou Y. Investigation on subcellular localization of Rice stripe virus in its vector small brown planthopper by electron microscopy. Virol J. 2013; 10: 310 10.1186/1743-422X-10-310 . 24139455 PMC4015704

[56] Qin FL, Liu WW, Wu N, Zhang L, Zhang ZK, Zhou XP, et al Invasion of midgut epithelial cells by a persistently transmitted virus is mediated by sugar transporter 6 in its insect vector. PLoS Pathog. 2018; 14: e1007201 10.1371/journal.ppat.1007201 . 30052679 PMC6082570

[57] Overby AK, Pettersson RF, Grunewald K, Huiskonen JT. Insights into bunyavirus architecture from electron cryotomography of Uukuniemi virus. Proc Natl Acad Sci U S A. 2008; 105: 2375–9. 10.1073/pnas.0708738105 . 18272496 PMC2268144

[58] Schelhaas M. Come in and take your coat off—how host cells provide endocytosis for virus entry. Cell Microbiol. 2010; 12: 1378–88. 10.1111/j.1462-5822.2010.01510.x . 20678171

[59] Guardado-Calvo P, Rey FA. The Envelope Proteins of the Bunyavirales. Adv Virus Res. 2017; 98: 83–118. 10.1016/bs.aivir.2017.02.002 . 28433053

[60] Kielian M. Mechanisms of Virus Membrane Fusion Proteins. Annu Rev Virol. 2014; 1:171–89. 10.1146/annurev-virology-031413-085521 . 26958720

[61] Guardado-Calvo P, Bignon EA, Stettner E, Jeffers SA, Perez-Vargas J, Pehau-Arnaudet G, et al Mechanistic Insight into Bunyavirus-Induced Membrane Fusion from Structure-Function Analyses of the Hantavirus Envelope Glycoprotein Gc. PLoS pathog. 2016; 12: e1005813 10.1371/journal.ppat.1005813 . 27783711 PMC5082683

[62] de Boer SM, Kortekaas J, Spel L, Rottier PJM, Moormann RJM, Bosch BJ. Acid-Activated Structural Reorganization of the Rift Valley Fever Virus Gc Fusion Protein. J Virol. 2012; 86: 13642–52. 10.1128/JVI.01973-12 . 23035232 PMC3503025

[63] Tani H, Shimojima M, Fukushi S, Yoshikawa T, Fukuma A, Taniguchi S, et al Characterization of Glycoprotein-Mediated Entry of Severe Fever with Thrombocytopenia Syndrome Virus. J Virol. 2016; 90: 5292–301. 10.1128/JVI.00110-16 . 26984731 PMC4934762

[64] Dessau M, Modis Y. Crystal structure of glycoprotein C from Rift Valley fever virus. Proc Natl Acad Sci U S A. 2013; 110: 1696–701. 10.1073/pnas.1217780110 . 23319635 PMC3562824

[65] Willensky S, Bar-Rogovsky H, Bignon EA, Tischler ND, Modis Y, Dessau M. Crystal Structure of Glycoprotein C from a Hantavirus in the Post-fusion Conformation. PLoS Pathog. 2016; 12: e1005948 10.1371/journal.ppat.1005948 . 27783673 PMC5081248

[66] Tischler ND, Gonzalez A, Perez-Acle T, Rosemblatt M, Valenzuela PDT. Hantavirus Gc glycoprotein: evidence for a class II fusion protein. J Gen Virol. 2005; 86: 2937–47. 10.1099/vir.0.81083-0 . 16227214

[67] Li S, Wang S, Wang X, Li X, Zi J, Ge S, et al Rice stripe virus affects the viability of its vector offspring by changing developmental gene expression in embryos. Sci Rep. 2015; 5:7883 10.1038/srep07883 . 25601039 PMC4298728

[68] Kost TA, Condreay JP, Jarvis DL. Baculovirus as versatile vectors for protein expression in insect and mammalian cells. Nat Biotechnol. 2005; 23: 567–75. 10.1038/nbt1095 . 15877075 PMC3610534

[69] Zhu M, Jiang L, Bai BH, Zhao WY, Chen XJ, Li J, et al The intracellular immune receptor sw-5b confers broad-spectrum resistance to tospoviruses through recognition of a conserved 21-amino acid viral effector epitope. Plant Cell. 2017; 29: 2214–32. 10.1105/tpc.17.00180 . 28814646 PMC5635987

[70] Kong L, Qiu XF, Kang JG, Wang Y, Chen H, Huang J, et al A Phytophthora Effector Manipulates Host Histone Acetylation and Reprograms Defense Gene Expression to Promote Infection. Curr Biol. 2017; 27: 981–91. 10.1016/j.cub.2017.02.044 . 28318979

[71] Ma ZC, Zhu L, Song TQ, Wang Y, Zhang Q, Xia YQ, et al A paralogous decoy protects Phytophthora sojae apoplastic effector PsXEG1 from a host inhibitor. Science. 2017; 355: 710–4. 10.1126/science.aai7919 . 28082413

[72] Li J, Feng Z, Wu J, Huang Y, Lu G, Zhu M, et al Structure and function analysis of nucleocapsid protein of tomato spotted wilt virus interacting with RNA using homology modeling. J Biol Chem. 2015; 290: 3950–61. 10.1074/jbc.M114.604678 . 25540203 PMC4326804

[73] Lu G, Li J, Zhou YJ, Zhou XP, Tao XR. Model-based structural and functional characterization of the Rice stripe tenuivirus nucleocapsid protein interacting with viral genomic RNA. Virology. 2017; 506: 73–83. 10.1016/j.virol.2017.03.010 . 28359901

