# Supplementary figures and images for: Tenuivirus utilizes its glycoprotein as a helper component to overcome insect midgut barriers for its circulative and propagative transmission
Source: PLoS Pathog. 2019 Mar 28;15(3):e1007655. doi: 10.1371/journal.ppat.1007655 (PMC6456217; doi:10.1371/journal.ppat.1007655)

**A**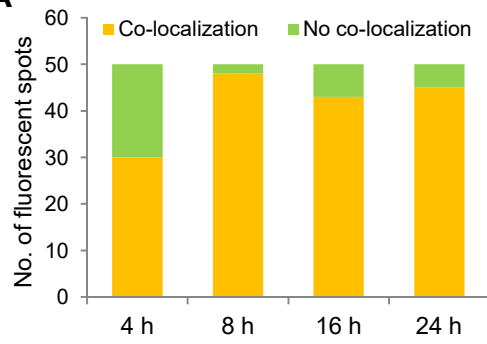**B**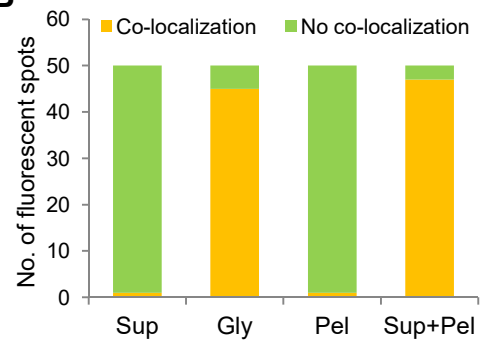

Supplement: S1 Fig — (A) Quantification of co-localization of RSV virions and NSvc2 in SBPH midgut at 4, 8, 16 and 24 h post feeding on RSV-infected rice plants. (B) Quantification of co-localization of RSV virions and NSvc2 in SBPH midgut after feeding on different centrifugation fractions. Fifty fluorescent spots were randomly selected from 30 guts per experiment and analyzed for co-localization of RSV virions and NSvc2. Sup, supernatant fractions; Gly, glycerol fractions; Pel, resuspended pellet sample. (PDF) [file ppat.1007655.s001.pdf]

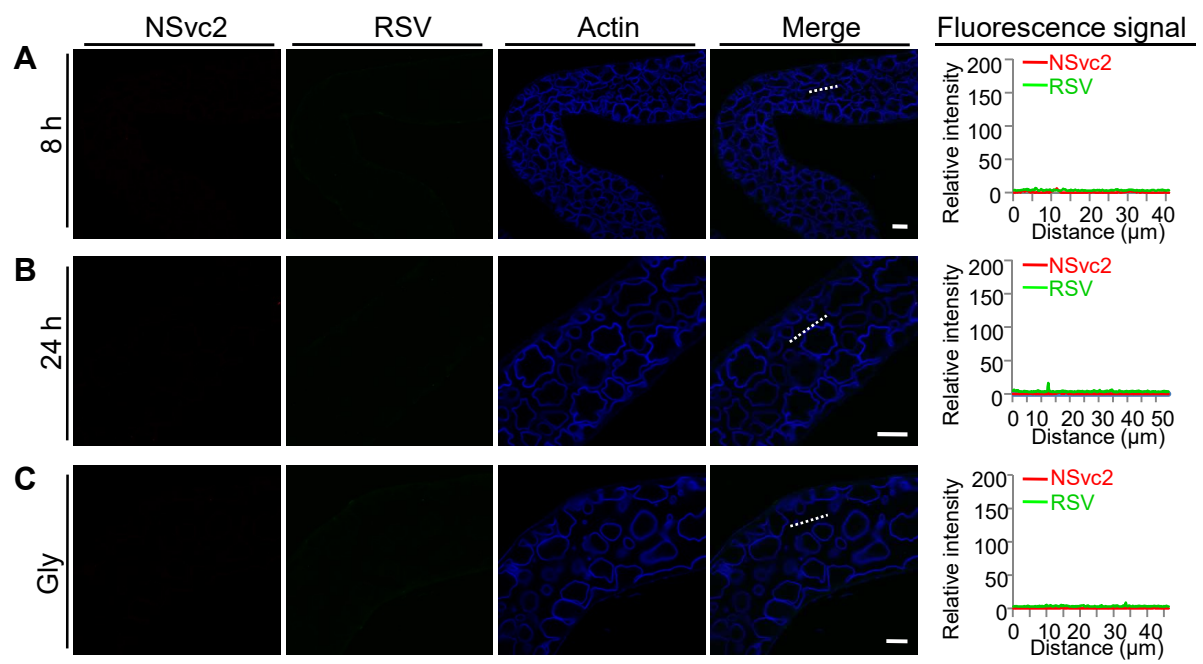

Supplement: S2 Fig — (A and B) Immunofluorescence labeling of NSvc2 and RSV virions on the surface of SBPH intestinal microvillus (blue) at 8 h (A) and 24 h (B) post feeding on the healthy rice plants. (C) Immunofluorescence labeling of NSvc2 and RSV virions on the surface of SBPH intestinal microvillus at 24 h post feeding on the mixed glycerol fractions prepared from the healthy rice plants. The samples were probed with the NSvc2-N (red), RSV NP (green), or actin (blue) specific antibody. Bar, 25 μm. The overlap fluorescence spectra from NSvc2 and RSV virion labelings at different stages were determined using the white dashed line and shown right. (PDF) [file ppat.1007655.s002.pdf]

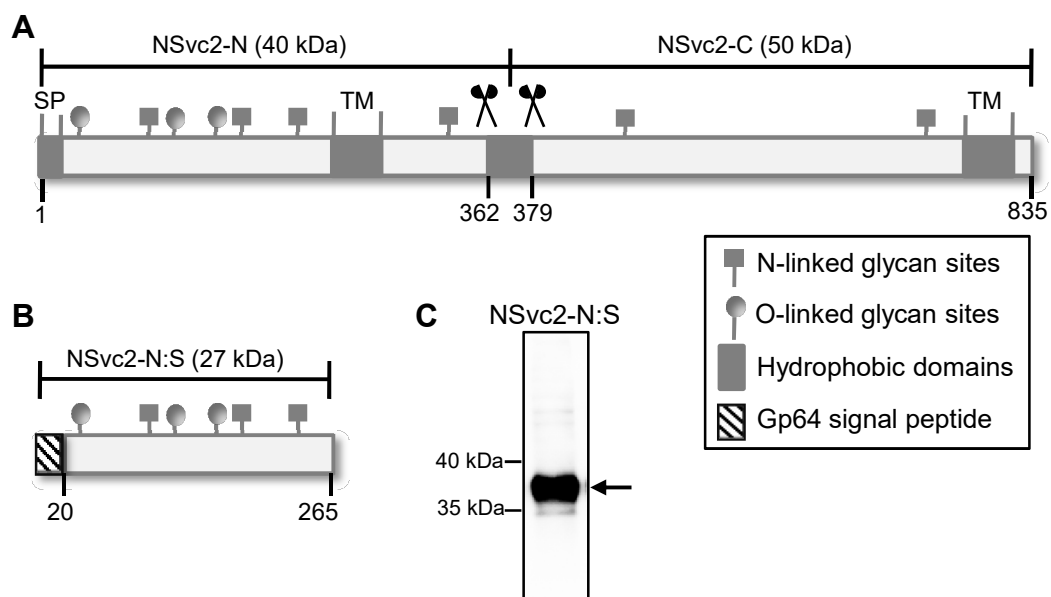

Supplement: S3 Fig — (A) A diagram of NSvc2 with different domains and putative glycosylation sites. SP, signal peptide; TM, transmembrane domain. (B) A diagram of NSvc2-N:S with different domains and putative glycosylation sites. The signal peptide of NSvc2-N:S is replaced with a Gp64 signal peptide. (C) Detection of NSvc2-N:S expression in Sf9 cells using a NSvc2-N specific antibody. Protein marker sizes are indicated on the left side and the labeled NSvc2-N:S band is indicated with an arrow. (PDF) [file ppat.1007655.s003.pdf]

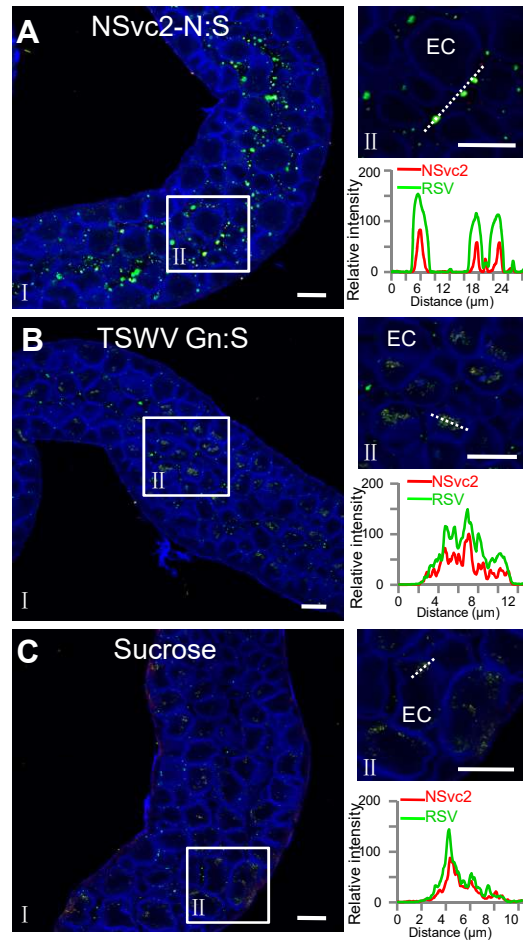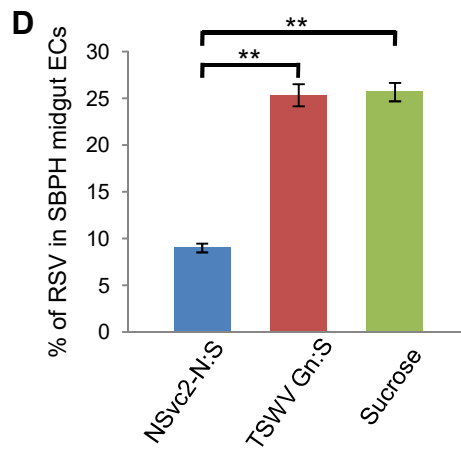

Supplement: S4 Fig — (A-C) Effects of pre-feeding with purified NSvc2-N:S (A), TSWV Gn:S (B) and sucrose alone (C) on RSV virion entrance into SBPH midguts. The boxed regions are enlarged and shown on the right side. The overlap fluorescence spectra were from the white dashed line indicated areas. (D) Percentages of RSV virion invaded SBPH midgut epithelial cells. **, p < 0.01 by the student t-test. EC, epithelial cells; Bar, 25 μm. (PDF) [file ppat.1007655.s004.pdf]

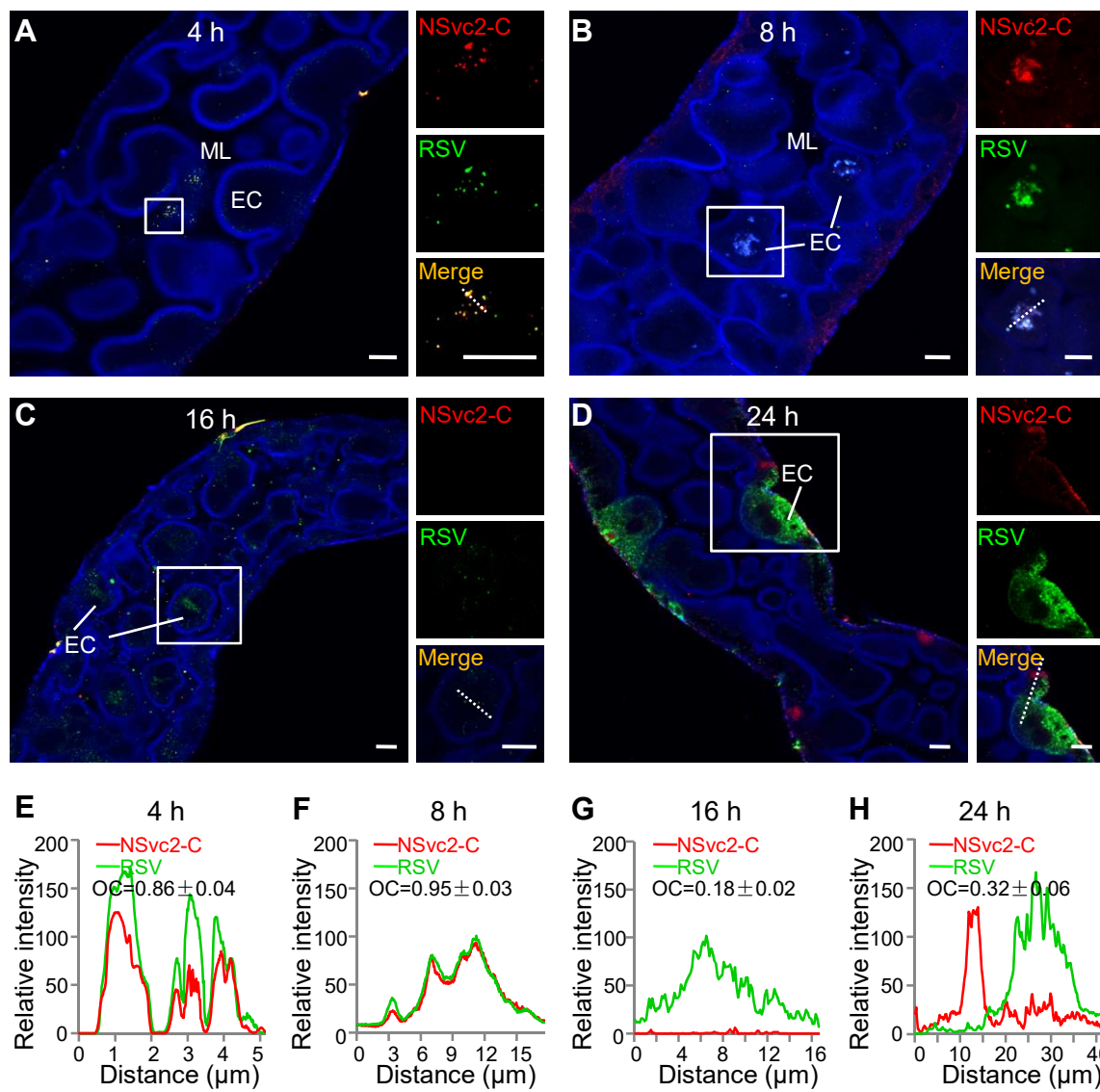

Supplement: S5 Fig — (A) NSvc2-C and RSV virions were co-localized on the surface of midgut microvillus (blue) at 4 h post feeding on the RSV-infected rice seedlings. The boxed region was enlarged and shown with three panels on the right side. The labeled NSvc2-C is shown in red and the labeled RSV virions are shown in green. (B) NSvc2-C and RSV virions were co-localized in the endosomal-like vesicles inside the midgut epithelial cells at 8 h post feeding. (C) RSV virions were detected in the cytosol of epithelial cells at 16 h post feeding but not NSvc2-C. (D) NSvc2-C was not detected in the cytosol with RSV virions at 24 h post feeding. (E–H) Analyses of overlap fluorescence spectra from the white dashed line indicated regions in the merged images. The overlap coefficient (OC) values were determined using the LAS X software. ML, midgut lumen; EC, epithelial cells; Bar, 10 μm. (PDF) [file ppat.1007655.s005.pdf]

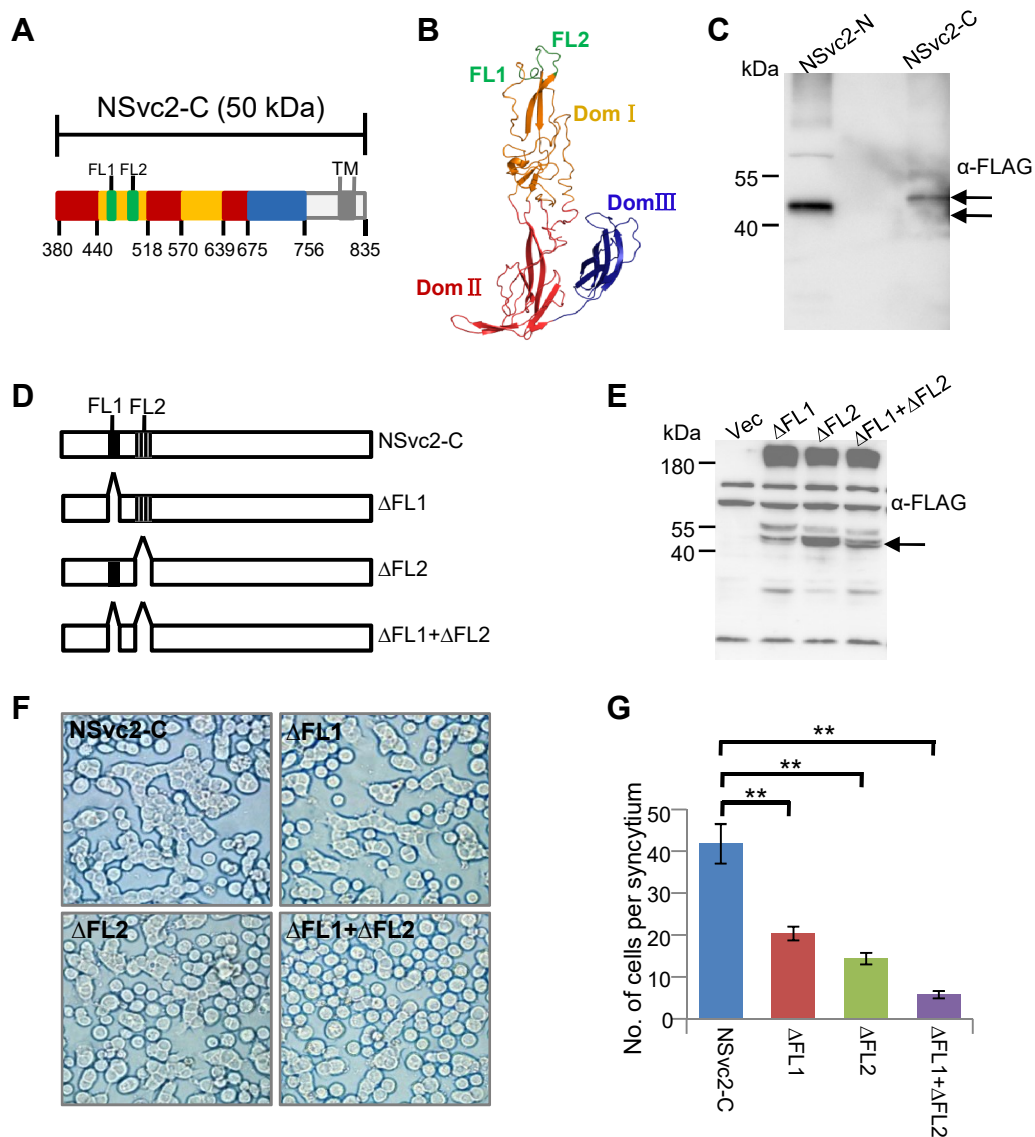

Supplement: S6 Fig — (A) A diagram showing different domains in NSvc2-C. Domain I is in yellow, domain II is in red and domain III is in blue. Fusion loops (FL1 and FL2) are shown in green and a hydrophobic region in gray. (B) A three-dimensional homology structure model of NSvc2-C, with the same color arrangement as shown in (A). (C) Expressions of the FLAG-tagged NSvc2-N or NSvc2-C in the Sf9 cells were confirmed by Western blot assays. The NSvc2-N-FLAG and NSvc2-C-FLAG proteins were enriched individually and then detected using an anti-FLAG antibody. Arrows indicate the bands of the expressed NSvc2-N-FLAG (40 kDa) or NSvc2-C-FLAG (50 kDa) proteins. (D) Schematic representations of NSvc2-C and its FL deletion mutants. Locations of FL1 (black) and FL2 (gray) are shown. Deletions of one fusion loop (ΔFL1 or ΔFL2) or both fusion loops (ΔFL1+ΔFL2) are indicated with upward open arrows. (E) Expressions of the FLAG-tagged NSvc2-C and its FL deletion mutants in the Sf9 cells were determined by immunoblotting. The blots were probed with a FLAG tag-specific antibody. Arrow indicates the bands of the expressed proteins. The empty vector (Vec) was used as a negative control. (F and G) Analyses of fusogenic activities of NSvc2-C and its FL deletion mutants. Sf9 cells were infected with the recombinant baculoviruses expressing NSvc2-C or one of its deletion mutants. At 48 h post infection, the Sf9 cells were treated for membrane fusion assays (F). The numbers of cells per syncytium were counted and analyzed (G). The experiment was repeated three times. **, p < 0.01 by the student t-test. (PDF) [file ppat.1007655.s006.pdf]
