# Supplementary material for: Tenuivirus utilizes its glycoprotein as a helper component to overcome insect midgut barriers for its circulative and propagative transmission
Source: PLoS Pathog. 2019 Mar 28;15(3):e1007655. doi: 10.1371/journal.ppat.1007655 (PMC6456217; doi:10.1371/journal.ppat.1007655)
Supplement: S1 Table — (DOCX) [file ppat.1007655.s007.docx]

**S1 Table. RSV acquisition and transmission efficiency by SBPHs fed on the combined supernatant fractions (Sup), combined glycerol fractions (Gly), the resuspended pellet (Pel) sample or a combination of Sup and Pel.**

| **Feeding Solution** | **RSV acquisition ^a^** | | | **Virus transmission ^b^** | | |
| --- | --- | --- | --- | --- | --- | --- |
|  | **Ⅰ^c^** | **Ⅱ** | **Ⅲ** | **Ⅰ** | **Ⅱ** | **Ⅲ** |
| Sup | 0%  (0/100) | 0%  (0/100) | 0%  (0/100) | 0%  (0/98) | 0%  (0/99) | 0%  (0/97) |
| Gly | 16%  (16/100) | 20%  (20/100) | 14%  (14/100) | 4%  (4/97) | 6%  (6/99) | 3%  (3/97) |
| Pel | 0%  (0/100) | 0%  (0/100) | 0%  (0/100) | 0%  (0/99) | 0%  (0/97) | 0%  (0/99) |
| Sup+Pel | 23%  (23/100) | 18%  (18/100) | 20%  (20/100) | 7%  (7/98) | 4%  (4/98) | 5%  (5/99) |

^a^ No. of RSV-infected/Total number of SBPHs tested.

^b^ No. of RSV-infected/Total number of rice seedlings tested.

^c^ Biological repeat.
