# Supplementary material for: Tenuivirus utilizes its glycoprotein as a helper component to overcome insect midgut barriers for its circulative and propagative transmission
Source: PLoS Pathog. 2019 Mar 28;15(3):e1007655. doi: 10.1371/journal.ppat.1007655 (PMC6456217; doi:10.1371/journal.ppat.1007655)
Supplement: S2 Table — (DOCX) [file ppat.1007655.s008.docx]

**S2 Table. RSV acquisition and transmission efficiency by SBPHs pre-fed with NSvc2-N:S or its mutant proteins followed by feedings on the RSV-infected rice seedlings.**

| **Pre-fed solution** | **RSV acquisition ^a^** | | | **Virus transmission ^b^** | | |
| --- | --- | --- | --- | --- | --- | --- |
|  | **Ⅰ^c^** | **Ⅱ** | **Ⅲ** | **Ⅰ** | **Ⅱ** | **Ⅲ** |
| NSvc2-N:S | 25%  (25/100) | 16%  (16/100) | 20%  (20/100) | 7%  (7/99) | 4%  (4/99) | 6%  (6/98) |
| NSvc2-N:S^N114A/N199A/N232A^ | 42% (42/100) | 32% (32/100) | 38% (38/100) | 16% (16/97) | 11% (11/98) | 15%  (15/98) |
| NSvc2-N:S^S38A/S128A/S183A^ | 22/100 (22%) | 28/100 (28%) | 18/100 (18%) | 5/97  (5%) | 7/99  (7%) | 4/98  (4%) |
| TSWV Gn:S | 47% (47/100) | 55% (55/100) | 45% (45/100) | 15% (15/99) | 22% (22/98) | 13%  (13/97) |
| Sucrose | 48% (48/100) | 52% (52/100) | 46% (46/100) | 20% (19/96) | 18% (18/97) | 15%  (15/98) |

^a^ No. of RSV-infected/Total number of SBPHs tested.

^b^ No. of RSV-infected/Total number of rice seedlings tested.

^c^ Biological repeat.
