# Supplementary material for: Tenuivirus utilizes its glycoprotein as a helper component to overcome insect midgut barriers for its circulative and propagative transmission
Source: PLoS Pathog. 2019 Mar 28;15(3):e1007655. doi: 10.1371/journal.ppat.1007655 (PMC6456217; doi:10.1371/journal.ppat.1007655)
Supplement: S3 Table — (DOCX) [file ppat.1007655.s009.docx]

**S3 Table. RSV acquisition and transmission efficiency by SBPHs fed with a mixture of purified RSV virions and NSvc2, or a mixture of purified RSV virions and an NSvc2 mutant.**

| **Feed glycoprotein and RSV virions**  **simultaneously** | **RSV acquisition ^a^** | | | **Virus transmission ^b^** | | |
| --- | --- | --- | --- | --- | --- | --- |
|  | **Ⅰ^c^** | **Ⅱ** | **Ⅲ** | **Ⅰ** | **Ⅱ** | **Ⅲ** |
| NSvc2  (Wild type) | 15% (15/100) | 18% (18/100) | 12% (12/100) | 5% (5/99) | 5% (5/97) | 4% (4/99) |
| NSvc2  (N114A/N199A/N232A) | 0% (0/100) | 0% (0/100) | 0% (0/100) | 0%  (0/99) | 0% (0/100) | 0% (0/98) |
| NSvc2  (F460A/F489A/Y498A) | 3% (3/100) | 3% (3/100) | 2% (2/100) | 0% (0/98) | 1% (1/97) | 0% (0/98) |

^a^ No. of RSV-infected/Total number of SBPHs tested.

^b^ No. of RSV-infected/Total number of rice seedlings tested.

^c^ Biological repeat.
