# Supplementary material for: Tenuivirus utilizes its glycoprotein as a helper component to overcome insect midgut barriers for its circulative and propagative transmission
Source: PLoS Pathog. 2019 Mar 28;15(3):e1007655. doi: 10.1371/journal.ppat.1007655 (PMC6456217; doi:10.1371/journal.ppat.1007655)
Supplement: S4 Table — (DOCX) [file ppat.1007655.s010.docx]

**S4 Table.** **RSV acquisition and transmission efficiency by SBPHs pre-fed with NSvc2 or one of its mutants followed by feedings on purified RSV virions.**

| **Sequentially feed glycoprotein and RSV virions** | **RSV acquisition ^a^** | | | **Virus transmission ^b^** | | |
| --- | --- | --- | --- | --- | --- | --- |
|  | **Ⅰ^c^** | **Ⅱ** | **Ⅲ** | **Ⅰ** | **Ⅱ** | **Ⅲ** |
| NSvc2  (Wild type) | 18% (18/100) | 20% (20/100) | 22% (22/100) | 7% (7/99) | 6% (6/99) | 8% (8/98) |
| NSvc2  (N114A/N199A/N232A) | 0% (0/100) | 0% (0/100) | 0% (0/100) | 0% (0/98) | 0% (0/97) | 0% (0/99) |
| NSvc2  (F460A/F489A/Y498A) | 4% (4/100) | 5% (5/100) | 4% (4/100) | 1% (1/98) | 2% (2/99) | 2% (2/98) |
| NSvc2-N:S | 0% (0/100) | 0% (0/100) | 0% (0/100) | 0% (0/99) | 0% (0/98) | 0% (0/99) |

^a^ No. of RSV-infected/Total number of SBPHs tested.

^b^ No. of RSV-infected/Total number of rice seedlings tested.

^c^ Biological repeat.
