# Supplementary material for: Tenuivirus utilizes its glycoprotein as a helper component to overcome insect midgut barriers for its circulative and propagative transmission
Source: PLoS Pathog. 2019 Mar 28;15(3):e1007655. doi: 10.1371/journal.ppat.1007655 (PMC6456217; doi:10.1371/journal.ppat.1007655)
Supplement: S5 Table — (DOCX) [file ppat.1007655.s011.docx]

**S5 Table. RSV acquisition and transmission efficiency by SBPHs micro-injected with the combined glycerol fractions, the resuspended pellet sample or sucrose solution.**

| **Micro-injecting samples** | **RSV acquisition ^a^** | | | **Virus transmission ^b^** | | |
| --- | --- | --- | --- | --- | --- | --- |
|  | **Ⅰ^c^** | **Ⅱ** | **Ⅲ** | **Ⅰ** | **Ⅱ** | **Ⅲ** |
| Pel (70 ng/μl) | 66% (58/87) | 72% (67/93) | 70% (63/90) | 22% (19/85) | 27% (25/91) | 27% (24/90) |
| Gly (20 ng/μl) | 40% (36/90) | 37% (33/89) | 45% (42/92) | 17% (15/89) | 14% (12/87) | 15% (14/91) |
| Buffer (0 ng/μl) | 0% (0/75) | 0% (0/80) | 0% (0/65) | 0%  (0/74) | 0%  (0/79) | 0%  (0/63) |

^a^ No. of RSV-infected/Total number of SBPHs tested.

^b^ No. of RSV-infected/Total number of rice seedlings tested.

^c^ Biological repeat.
